# Inflammasome-Independent NLRP3 Restriction of a Protective Early Neutrophil Response to Pulmonary Tularemia

**DOI:** 10.1371/journal.ppat.1006059

**Published:** 2016-12-07

**Authors:** Sivakumar Periasamy, Hongnga T. Le, Ellen B. Duffy, Heather Chin, Jonathan A. Harton

**Affiliations:** Department of Immunology and Microbial Disease, Albany Medical College, Albany, New York, United States of America; Emory University School of Medicine, UNITED STATES

## Abstract

*Francisella tularensis* (Ft) causes a frequently fatal, acute necrotic pneumonia in humans and animals. Following lethal Ft infection in mice, infiltration of the lungs by predominantly immature myeloid cells and subsequent myeloid cell death drive pathogenesis and host mortality. However, following sub-lethal Ft challenge, more mature myeloid cells are elicited and are protective. In addition, inflammasome-dependent IL-1β and IL-18 are important for protection. As Nlrp3 appears dispensable for resistance to infection with *Francisella novicida*, we considered its role during infection with the virulent Type A strain SchuS4 and the attenuated Type B live vaccine strain LVS. Here we show that both *in vitro* macrophage and *in vivo* IL-1β and IL-18 responses to Ft LVS and SchuS4 involve both the Aim2 and Nlrp3 inflammasomes. However, following lethal infection with Francisella, IL-1r-, Caspase-1/11-, Asc- and Aim2-deficient mice exhibited increased susceptibility as expected, while Nlrp3-deficient mice were more resistant. Despite reduced levels of IL-1β and IL-18, in the absence of Nlrp3, Ft infected mice have dramatically reduced lung pathology, diminished recruitment and death of immature myeloid cells, and reduced bacterial burden in comparison to wildtype and inflammasome-deficient mice. Further, increased numbers of mature neutrophil appear in the lung early during lethal Ft infection in Nlrp3-deficient mice. Finally, Ft infection induces myeloid and lung stromal cell death that in part requires Nlrp3, is necrotic/necroptotic in nature, and drives host mortality. Thus, Nlrp3 mediates an inflammasome-independent process that restricts the appearance of protective mature neutrophils and promotes lethal necrotic lung pathology.

## Author Summary

The Nlrp3 inflammasome is critical for various innate and adaptive immune responses through elaboration of IL-1β and IL-18. In contrast to the anticipated minimal, or perhaps absent, role of Nlrp3 in the pathogenesis of pulmonary tularemia, we find that Nlrp3 is a host susceptibility factor. Likely through promoting necrotic/necroptotic cell death, Nlrp3 contributes to the immature myeloid response and necrotic pathology that characterize lethal infection with Francisella tularensis.

## Introduction

Pulmonary tularemia is an acute, necrotizing, and highly lethal pneumonia caused by the highly pathogenic zoonotic bacterium *Francisella tularensis* (Ft) [1]. The Type A (*F*. *tularensis tularensis*) and Type B (*F*. *tularensis holarctica*) strains cause disease in both animals and humans [2]. Type A strains (e.g. SchuS4) are highly pathogenic to humans and animals and inhalation of as few as 10 cfu of SchuS4 causes lethal disease in humans and mice [1]. Thus, Type A strains are classified as category ‘A’ biothreat agents by the CDC [3]). Although used to model pulmonary tularemia in mice, the attenuated Type B live vaccine strain (Ft LVS) is not pathogenic to humans. Another strain, *Francisella novicida* (Fn) is closely related to Ft and highly pathogenic in rodents, but nonpathogenic in humans [3].

During lethal pulmonary tularemia, Ft infects lung phagocytes and replicate intracellularly [1–2]. Instead of eliciting effective innate immune responses capable of controlling bacteria, immature myeloid cells/myeloid-suppressor cells are recruited [4]. These immature cells are ineffective phagocytes, but prone to necrosis resulting in necrotic lung damage and subsequent death of mice. In contrast, during sublethal infection, infiltrating mature neutrophils and inflammatory monocytes/macrophages outnumber immature myeloid cells and are essential for protection of surviving mice. Thus, the necrotizing inflammation and extensive tissue damage associated with lethal disease during pulmonary tularemia can be attributed to this dysregulated myeloid cell response [4]. How these immature myeloid cells are recruited, how they die, and how dying cells result in lung pathology during pulmonary tularemia is not known. Previous studies have suggested apoptosis as a mode of myeloid cell death through active Caspase-3 in myeloid cells in the spleen, liver, and lungs of Type A strain KU49 infected mice [5, 6]. In contrast, another study reported that activated Caspase-3 or AnnexinV expression was rarely observed at 3 days post-infection in lungs of mice infected with SchuS4 [7]. However, while we observed that Ft induces necrotic changes in myeloid cells including immature cells in the lungs [4], how these cells die and how that death contributes to lethal lung damage is unknown.

Although production of the pro-inflammatory cytokines IL-1β, IL-6 and TNFα is delayed during tularemia [8–10], mice deficient for these cytokines or the relevant receptors are more susceptible to Ft infection [11–13]. Previous studies have also clearly shown a protective role for IL-1β in mice during Fn infection [14–16]. A recent study examined intranasal Ft LVS infection of IL-1^-/-^ and IL-18^-/-^ mice, revealing increased susceptibility of IL-18 deficient mice and a critical role for IL-1β in the early production of protective anti-Ft LPS IgM by B1a B cells [13]. These studies suggest that early inflammatory cytokine responses, such as that of IL-1β and IL-18 are important for survival.

Several studies have investigated the protective role of the Aim2/Asc/Caspase-1 inflammasome axis in resistance to subcutaneous infection with Fn [14–19]. Aim2 binds dsDNA which assembles an Aim2 inflammasome via oligomerization of ASC and recruitment/activation of proCaspase-1 to enzymatically process proIL-1β and proIL-18 [17, 20, 21]. The Aim2 inflammasome also promotes Caspase-1-dependent cell death (pyroptosis) [17, 20]. Indeed, recognition of Fn dsDNA by Aim2 appears solely responsible for Fn elicited inflammasome activation as mouse Nlrp1, Nlrp3, and Nlrc4 do not respond to Fn [14, 15]. In contrast, in human cells both Aim2 and NLRP3 inflammasomes respond to Fn and Ft LVS [22]. NLRP3 also seeds inflammasome formation, but is activated by a wide array of stimuli and likewise can promote pyroptotic and Asc-dependent, but Caspase-1-independent (pyronecrotic) death of myeloid cells during infection [21, 23–26]. Although two Ft LVS studies using the LVS mutants LVSΔripA and FTL-0325 report that IL-1β responses enhanced by these mutants are independent of Nlrp3 [27, 28], whether Nlrp3 is involved in the inflammasome response to Ft LVS or SchuS4 in mice is unclear. However, the *in vivo* response of Nlrp3^-/-^ mice to infection with the Ft LVS or SchuS4 strain, most relevant to human disease, is essentially unexplored.

Here, we report that Nlrp3^-/-^ mice exhibit resistance to Ft infection through mature myeloid cell response in lungs and decreased myeloid and lung cell death during pulmonary tularemia. Consistent with previous reports, IL-1r^-/-^, Asc^-/-^, Casp-1/11^-/-^, and Aim2^-/-^ mice were more susceptible to Ft infection, but a significant proportion of Nlrp3^-/-^ mice survive. Despite limited IL-1β and IL-18 production, Ft-infected Nlrp3^-/-^ mice had reduced lung pathology, lower bacterial burden, and fewer dead lung myeloid and stromal cells when compared to wildtype and inflammasome-deficient mice. A mature population of neutrophils appearing in the lung on day 1 post-infection is necessary for protection. We also demonstrate that Ft-elicited cell death is likely due to a necrotic/necroptotic mechanism involving Nlrp3, but Asc/Caspase-1 inflammasome-independent. Our results suggest that while Asc and Caspase-1-mediated IL-1β and IL-18 play protective roles, Nlrp3 is a host susceptibility factor detrimental during Ft infection.

## Results

### *F*. *holarctica* (LVS) and *F*. *tularensis* (Schu S4) activate the Nlrp3 inflammasome

**Fig 1:**
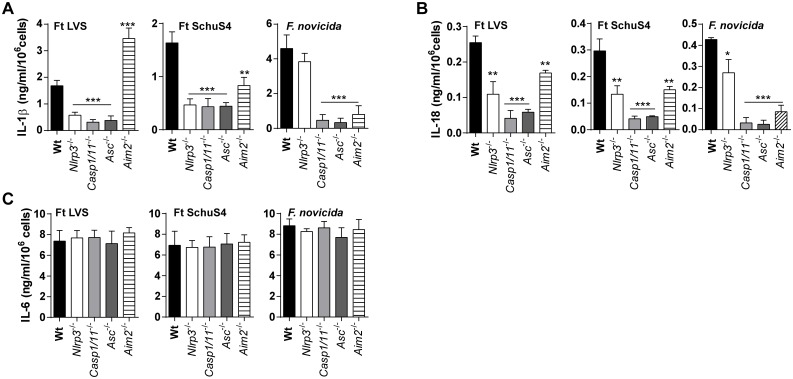
*F*. *tularensis* and *F*. *novicida* activate both Nlrp3- and Aim2-inflammasomes. (A-C) Levels of IL-1β, IL-18 or IL-6 measured in culture supernatants of BMDM infected with Ft LVS, SchuS4 or *F*. *novicida* at MOI = 100 for 24 h (mean ± SD of three independent experiments, Student’s t-test, *p<0.05, **p<0.01 or ***p<0.001 indicates the significant difference from wildtype BMDM).

A variety of gram negative bacteria activate the NLRP3 inflammasome [26]. However, it is well-established that protective innate immunity to *F*. *novicida* requires activation of the Aim2 inflammasome and that the Nlrp3 inflammasome is not required [14, 17–19, 29]. Further, elaboration of IL-1β by Fn infected macrophages requires neither Nlrp3 nor Nlrc4, while Asc is indispensable [29]. We previously reported that in human macrophages, both AIM2 and NLRP3 mediate the inflammasome response to *F*. *novicida* and Ft LVS [22]. Nevertheless, the role of Nlrp3 in Ft infection has not been well studied. Further, LVS and SchuS4 are the Francisella strains most relevant to epidemics of human tularemia [3]. To establish whether Nlrp3 is involved in the inflammasome response to LVS and SchuS4, BMDM from C57BL6J wildtype and Nlrp3^-/-^ mice were infected with Ft LVS, SchuS4 or Fn and their IL-1β and IL-18 responses measured. The corresponding responses of Casp1/11^-/-^, Asc^-/-^, and Aim2^-/-^ BMDM were evaluated as controls. As expected, IL-1β and IL-18 elaboration by these cells is dramatically reduced in the absence of Casp1/11 and Asc (Fig 1). Interestingly, IL-1β production was significantly reduced following Ft LVS or SchuS4 infection of cells from Nlrp3^-/-^ mice (Fig 1A). Curiously, macrophages from Aim2^-/-^ mice produced more IL-1β than wildtype cells in response to LVS infection but IL-1β production following SchuS4 infection was limited (Fig 1A). In contrast, IL-1β production following Fn infection was not reduced by deficiency in Nlrp3, but was abrogated without Aim2 as previously reported [17–19]. Surprisingly, the IL-18 response pattern differed. In the absence of Nlrp3 or Aim2, the macrophage IL-18 response to Ft LVS or SchuS4 infection was reduced by about 50% (Fig 1B). Infected Asc^-/-^ and Casp-1/11^-/-^ macrophages produced little IL-18 (Fig 1B). However, IL-18 processing by Nlrp3-deficient macrophages following Fn infection was significantly reduced, but still robust, while that of cells-deficient for Aim2, Asc or Casp-1/11 was similar to negative controls (Fig 1B). These observed changes in IL-1β and IL-18 are likely due to inflammasome-specific differences in the response to the bacterial strains, as other inflammasome-independent pro-inflammatory cytokines, including IL-6 (Fig 1C) and others (S1 Fig) were unaffected indicating that TLR responses are unaffected. Further, none of these genetic deficiencies altered macrophage infection by the Francisella strains used in this study, nor were proIL-1β protein levels substantially altered (S1 Fig). These results suggest that the specific Francisella strains differ in their utilization of the Nlrp3 and Aim2 inflammasomes for macrophage IL-1β and IL-18 responses. Further, Nlrp3 and Aim2 dependent LDH release was also observed for Ft LVS (S1 Fig). Collectively, our data reveal that Nlrp3 is responsive to Francisella strains other than Fn and thus Nlrp3 may have an important role in immunity and pathogensis of pulmonary tularemia.

### Nlrp3^-/-^ mice are less susceptible to lethal pulmonary Ft infection

**Fig 2:**
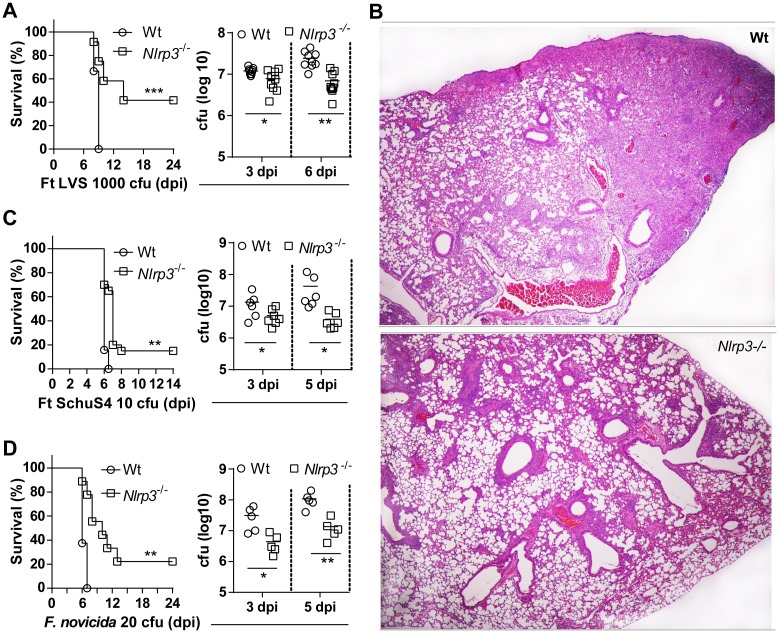
Nlrp3^-/-^ mice are less susceptible to lethal pulmonary Ft infection. (A) Survival of mice following lethal Ft LVS (1000 cfu) infection (% survival of three independent experiments, n = 18 mice, Log-rank (Mantel-Cox) test, ***p<0.001) and lung bacterial burden (mean ± SD of three independent experiments, n = 9 mice, Student’s t-test, *p<0.05). (B) Histological section of lung from wildtype mice infected with Ft LVS shows inflammatory foci and massive necrosis (‘necrotizing inflammation’) at 6 dpi, while these pathological changes were less extensive in lung section of Nlrp3^-/-^ mice (HE, 400x). (C) Survival of mice following Ft SchuS4 (10 cfu) infection (% survival of three independent experiments, n = 15 mice, Log-rank test, *p<0.05) and lung bacterial burden (mean ± SD of two independent experiments, n = 6, Student’s t-test, *p<0.05). (D) Survival of mice following lethal *F*. *novicida* (20 cfu) infection (% survival of two independent experiments, n = 12 mice, Log-rank test, *p<0.05) and lung bacterial burden (mean ± SD, n = 5 mice, Student’s t-test, *p<0.05).

The Aim2 inflammasome response is critical for resistance to intradermal infection with *F*. *novicida* [14–19]. Further, IL-1β and IL-18 are also important for resistance to pulmonary challenge with LVS [13]. Since Nlrp3 is required for LVS and SchuS4 elicited IL-1β/IL-18 response, we considered whether Nlrp3 is important for resistance to pulmonary Ft infection. Wildtype and Nlrp3^-/-^ mice were infected intranasally with a lethal dose of Ft LVS (1000 cfu) and monitored for survival. All wildtype mice succumbed to lethal LVS infection between 8–10 days (Fig 2A). Surprisingly, a large percentage (~50%) of Nlrp3^-/-^ mice survived lethal LVS infection. Compared to lethally infected wildtype mice, Nlrp3^-/-^ mice had reduced bacterial burden in lungs (Fig 2A), spleen and liver (S2A Fig) that was evident at 3 dpi and became significant at 5 dpi. Approximately 15% of Nlrp3^-/-^ mice survived infection with a four-fold higher challenge dose, but all succumbed with a 20-fold higher challenge dose (S2B Fig). Thus, resistance to Ft LVS observed in Nlrp3^-/-^ mice is challenge dose-dependent. Periodic clinical observations including weight loss and decreased activity indicated that all the mice were infected with Ft (S2C Fig). Ft LVS-infected wildtype mice exhibit overt pathological changes characterized by necrotizing inflammation in lungs, spleen and liver that correlates with a loss of pulmonary function and death [4]. In the lungs, this inflammation is characterized by progressive mixed cellular infiltration, serous to fibrinous exudates with cellular debris, and necrosis culminating in loss of airway space and function. Consistent with increased survival, the lungs of Nlrp3^-/-^ mice exhibited less necrosis and more preserved airway space in comparison to wildtype mice (Fig 2B).

Remarkably, while all wildtype mice succumbed to infection with the highly virulent and clinically relevant Type A strain SchuS4 (10 cfu) by 6–7 days, a small, but significant proportion (20%) of Nlrp3^-/-^ mice survived (Fig 2C). Similar to LVS infection, lung bacterial burdens were significantly reduced in Nlrp3^-/-^ mice at 5 days post-SchuS4 infection (Fig 2C). However, upon challenge with a larger inoculum (150 cfu), all Nlrp3^-/-^ mice succumbed to infection (S2D Fig). Curiously, although previous studies have shown that Asc^-/-^, Casp-1/11^-/-^ and Aim2^-/-^ mice are susceptible to intradermal Fn infection [13, 17, 18, 29], a significant proportion of Nlrp3^-/-^ mice (25%) survived intranasal infection with a lethal dose of Fn (20 cfu) compared with wildtype mice which all died by 6–7 days (Fig 2D). As with LVS and SchuS4 infection, the lungs of Nlrp3^-/-^ mice displayed reduced bacterial burdens at 3 and 5 days post-Fn infection (Fig 2D). Together, these data demonstrate a *Francisella* strain-independent, detrimental role for Nlrp3 in the pathogenesis of pulmonary tularemia.

### NLRP3-mediated pathogenesis is inflammasome independent

**Fig 3:**
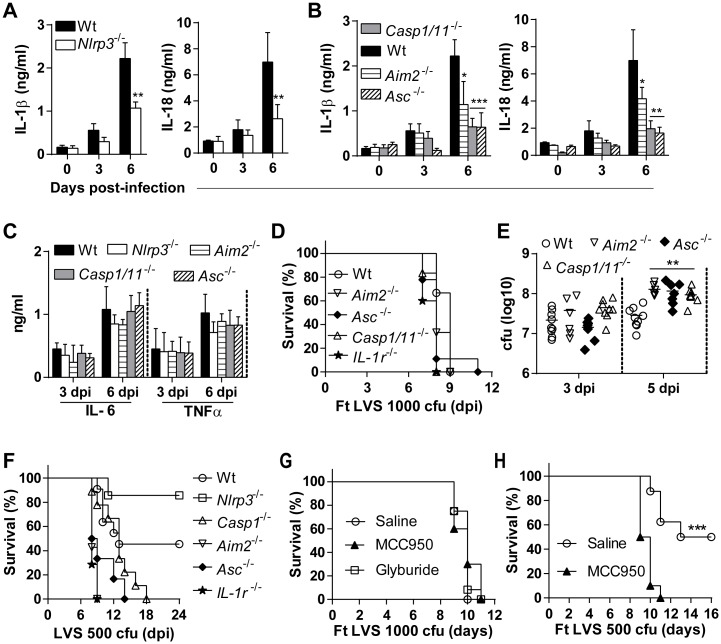
Nlrp3 mediates pathogenesis of pulmonary tularemia in an inflammasome-independent manner. (A) Levels of IL-1β and IL-18 measured in lung homogenates at indicated days post-Ft LVS infection (mean ± SD of two independent experiments, n = 6 mice, Student’s t-test, **p<0.01 indicates difference from wildtype mice). (B) Levels of IL-1β and IL-18 measured in lung homogenates at indicated days post-Ft LVS infection (mean ± SD of two independent experiments, n = 6 mice, Student’s t-test, *p<0.05 or ***p<0.001 indicates difference from wildtype mice). (C) Level of IL-6 and TNFα measured in lung homogenates at indicated days post-Ft LVS infection (mean ± SD of two independent experiments, n = 6 mice, Student’s t-test). (D) Survival of mice following lethal Ft LVS (1000 cfu) infection (% survival of two independent experiments, n = 12, Log-rank test). (E) Lung bacterial burden (mean ± SD of two independent experiments, n = 6 mice, Student’s t-test, **p<0.01). (F) Survival of mice following with sub-lethal Ft LVS (500 cfu) infection (% survival of two independent experiments, n = 12, Log-rank test). (G) Survival of mice following with lethal (1000 cfu) Ft LVS infection and treatment with MCC950 (250μg/mouse daily at 2–7 dpi) or with Glyburide (500μg/mouse daily at 2–6 dpi) by i.p route (% survival of two independent experiments, n = 12 mice, Log-rank test). (H) Survival of mice following with sub-lethal Ft LVS (500 cfu) infection and MCC950 treatment (250μg/mouse daily at 2–7 dpi) by i.p route (% survival of two independent experiments, n = 10 mice, Log-rank test).

IL-1 family cytokines mediate inflammatory processes essential for innate and adaptive immunity [30]. IL-1β and IL-18 are critical for protective immunity against subcutaneous Fn infection [14–16] as well as pulmonary Ft LVS infection [13]. Although Fn elicited IL-1β responses are Nlrp3-independent, we observed significant protection against pulmonary Fn infection in Nlrp3-deficienct mice. However, the diminished IL-1β/IL-18 response of Nlrp3-deficient macrophages after Ft infection suggests that this response could be significantly impaired in Nlrp3^-/-^ mice, yet sufficient to provide protection against *Francisella*. Alternatively, Nlrp3 might play an inflammasome-independent role in the pathogenesis of pulmonary tularemia in wildtype mice. Interestingly, levels of IL-1β and IL-18 in the lung are markedly reduced in Nlrp3^-/-^ mice with slower kinetics over the initial 6 days of infection with LVS (Fig 3A) or SchuS4 (S3A Fig) infection, but are not completely ablated. Lung IL-1β and IL-18 levels in Ft infected mice deficient in Casp-1/11, Asc, and Aim2 were largely comparable to those of Nlrp3^-/-^ mice, with the exception of Aim2^-/-^ mice which had more IL-18 in their lungs at 6 dpi (Fig 3B). Lung IL-6 and TNFα responses were similar in all the mouse strains compared to to those of wildtype mice following Ft LVS infection (Fig 3C) or SchuS4 infection (S3B Fig). Protection of Nlrp3^-/-^ mice despite greatly reduced IL-1β/IL-18 responses, similar to those of Casp-1/11^-/-^ mice, was unexpected. Further, these results are seemingly contradictory to reports demonstrating the importance of IL-1β and IL-18 for protection against Francisella. We therefore considered whether other inflammasome-deficient mice were similarly protected. Unlike Nlrp3-deficient mice, Aim2^-/-^, Caspase-1/11^-/-^, Asc^-/-^, and IL-1R^-/-^ mice died within 8–11 dpi after lethal Ft LVS infection (Fig 3D) or between 6 and 7 dpi after SchuS4 (S3C Fig). In addition, lung bacterial burdens in these mice were approximately twice those of wildtype mice at 5 dpi (Fig 3E), but bacterial loads in the spleen and liver were similar to wildtype (S3D Fig). Further, following intransal instillation of a 50% lethal dose of Ft LVS nearly all Nlrp3^-/-^ mice survived while all of the IL-1R^-/-^ or other inflammasome component-deficient mice succumbed to infection (Fig 3F). After Ft LVS infection IL-1β and IL-18 levels are similar between Aim2- and Nlrp3-deficient mice, yet mice deficient in Aim2, ASC, or caspase-1/11 do not reproduce the survival phenotype of Nlrp3 mice. These levels are insufficient to protect Aim2-deficient mice, and do not account for the increased survival of Nlrp3-deficient mice. Thus, the detrimental impact of Nlrp3 is unlikely to be inflammasome-dependent.

The Nlrp3 inflammasome inhibitor MCC950 specifically blocks Nlrp3:Asc interaction and downstream caspase-1 activation without impacting the Aim2 inflammasome [31]. Wildtype mice infected with a lethal dose of Ft LVS and treated with MCC950 were not protected (Fig 3G). However, MCC950 treatment of wildtype mice receiving a 50% lethal dose of Ft LVS resulted in a complete loss of protection (Fig 3H). Although these mice are Nlrp3-sufficient, inhibition of the Nlrp3 inflammasome results in a phenotype similar to mice lacking ASC and caspase-1. Collectively, these results demonstrate that the detrimental role of Nlrp3 during pulmonary Ft infection is independent of the inflammasome. Our results also suggest that the diminished levels of IL-1β and IL-18 in the lungs of Nlrp3^-/-^ mice may be sufficient to support their critical protective function during pulmonary tularemia.

### NLRP3 deficiency does not alter Ft-specific IgM antibody levels during tularemia

**Fig 4:**
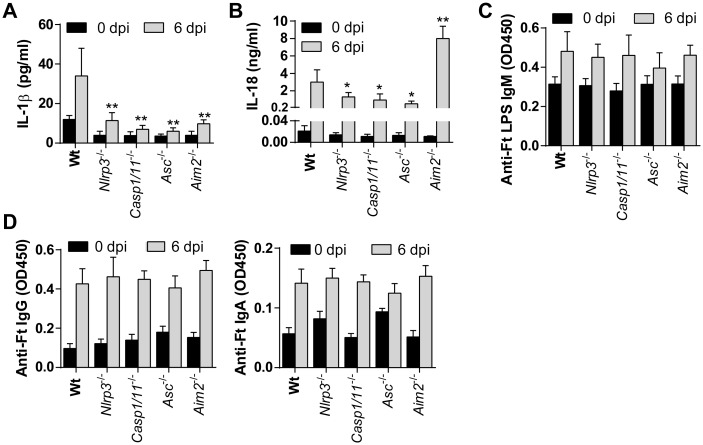
Innate antibody response is dispensable for protection during acute phase of tularemia. (A-B) Levels of IL-1β and IL-18 measured in serum (mean ± SD of two independent experiments, n = 6 mice, Student’s t-test, *p<0.05 or **p<0.01 indicates the difference from wildtype mice at 6 dpi). (C) Level of anti-Ft LPS IgM antibody titer measured in serum (mean ± SD of OD_450_ from two independent experiments, n = 6 mice, Student’s t-test). (D) Levels of anti-Ft IgG and IgA antibodies measured in serum (mean ± SD of OD_450_ from two independent experiments, n = 6, Student’s t-test).

A recent study reported that Ft-specific IgM produced by B1a B cells was significantly reduced in Il-1b^/-^, Il-1b^-/-^/Il-1a^-/-^, or Il-1r1^-/-^ mice compared to C57BL/6J wildtype or Il-1a^-/-^ mice and implicated as an explanation for susceptibility of IL-1β-deficient mice to pulmonary Ft LVS infection [13]. This study also demonstrated the importance of IL-18 for resistance to Ft infection [13]. Our results with Nlrp3-deficient mice appear to contradict these findings. However, while the serum level of IL-1β is reduced systemically in Nlrp3^-/-^, Asc^-/-^, Casp1/11^-/-^ and Aim2^-/-^ mice, IL-18 is only moderately reduced in Nlrp3^-/-^, Asc^-/-^ and Casp1/11^-/-^ mice, and is elevated in Aim2^-/-^ mice (Fig 4A and 4B). Although the serum IL-1β/IL-18 response follows a similar trend to that in the lungs, the magnitude of the systemic response is quite low by comparison (compare with Fig 3A and 3B). Further, no difference could be detected in the innate Ft LPS-specific IgM level (Fig 4C). Thus, reduced serum levels of IL-1β/IL-18 in mice deficient for inflammasome components does not appear to impact Ft LPS-specific IgM and does not correlate with their pattern of survival/mortality, suggesting that sufficient IL-1β/IL-18 is available to facilitate production of these antibodies. There was also no difference in IgG or IgA antibody levels in serum (Fig 4D) or BAL fluid (S4 Fig) of these mice at 6 dpi. As levels of anti-Ft LPS antibodies in serum and BAL fluid were comparable between Nlrp3-deficient and wildtype mice, it is unlikely that differences in the Ft-LPS-specific IgM antibody response play a critical role in the survival of Nlrp3-deficient mice.

### Nlrp3 prevents an early, protective neutrophil response during pulmonary tularemia

**Fig 5:**
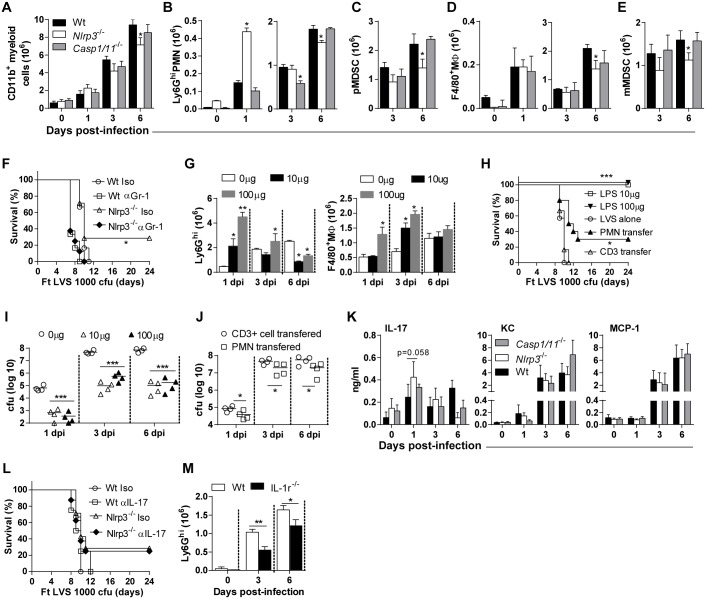
An early neutrophil response in Nlrp3^-/-^ mice is protective during pulmonary tularemia. (A-E) Total numbers of CD11b^+^ myeloid cells, Ly6G^+^ neutrophils, polymorphonucleated-MDSC (pMDSC), F4/80^+^macrophages or mono-nucleated-MDSC (mMDSC) in LVS-infected lungs (mean ± SD of two independent experiments, n = 6 mice, Student’s t-test, *p<0.05 indicates difference from wildtype mice at specific dpi). (F) Survival of mice following Ft LVS (1000 cfu) infection and treatment with anti-Gr-1 antibody (200μg/mouse, i.p route) at -1 and 1 dpi (% survival of two experiments, n = 6 mice, Log-rank test, *p<0.05). (G) Total numbers of Ly6G^high^ neutrophils in lungs of mice following i.n. instillation of LPS and infected 48 h later with Ft LVS (1000 cfu) (mean ± SD of three mice, Student’s t-test, *p<0.05 and **p<0.01 indicate the difference from those mice receiving no LPS but infected with LVS). (H) Survival of mice following Ft LVS (1000 cfu) infection. Naïve mice were first adoptively transferred with PMN (1 x10^6^ cells/mouse; isolated from bone marrow cells) or CD3+ T cells (1 x10^6^ cells/mouse; isolated from spleen) by intratracheal intubation and infected with Ft LVS on the following day. Other groups of naïve mice were treated with LPS (100 or 10 μg/mouse, i.n instillation) to elicit PMN/myeloid cell response, infected with Ft LVS after 48 h, and monitored for survival and mortality (% survival of two independent experiment, n = 10 mice, Log-rank test, *p<0.05 or ***p<0.001 indicates difference from naïve mice infected with LVS alone). (I) Lung bacterial burden in mice receiving i.n. instillation of LPS and infected 48 h later with Ft LVS (1000 cfu) (mean ± SD of four mice, Student’s t-test, *p<0.05 and **p<0.01 indicate the difference from those mice receiving no LPS but infected with LVS). (J) Lung bacterial burden in mice receiving PMN or CD3+ T cells post-infection with Ft LVS (1000 cfu) (mean ± SD of four mice, Student’s t-test, *p<0.05 and **p<0.01). (K) Levels of IL-17, KC and MCP-1 in lung homogenates (mean ± SD of two independent experiments, n = 6 mice, Student’s t-test). (L) Survival of mice following Ft LVS (1000 cfu) infection and treatment with anti-IL-17 antibody (200μg/mouse, i.p route) at 1 and 3 dpi (% survival of two independent experiments, n = 8 mice, Log-rank test). (M) Total numbers of Ly6G^high^ neutrophils in lungs of wildtype and IL-1r^-/-^ mice infected with lethal (1000 cfu) Ft LVS (mean ± SD of two independent experiments, n = 6 mice, Student’s t-test, *p<0.05 and **p<0.01).

During Ft infection, both myeloid (PMN and macrophages) and lymphoid (T and B cells) cells are thought important for protection [32–36]. However, we recently reported that necrotic lung damage and host death during lethal pulmonary tularemia is accompanied by predominating infiltration of the lung by death-prone immature myeloid cells with myeloid-derived suppressor cell (MDSC) phenotypes and function, specifically immature “band” neutrophils/PMN-MDSC (pMDSC) and monocytic-MDSC (mMDSC) [4]. Accordingly, Ly6G^hi^ neutrophils and F4/80^+^ macrophages capable of controlling bacteria are not prevalent in the lungs of lethally infected mice. We also showed that while eliciting mature neutrophils and macrophage is protective, neutrophils are essential [4]. Thus, the resistance of Nlrp3^-/-^ mice to Ft LVS might result from a change in the type or extent of the myeloid cell response. The total number of lung cells recovered and the frequencies of T and NK cells were similar between wildtype, Casp1/11^-/-^, and Nlrp3^-/-^ mice over the course of infection (S5 Fig). Interestingly,the number of lung CD11b+ myeloid cells in Nlrp3^-/-^ mice was also comparable to that in Casp1/11^-/-^ and wildtype mice until 6 dpi (Fig 5A), demonstrating that myeloid cell influx is largely unaffected. However, Ly6G^hi^ (i.e. mature) neutrophils were notably more abundant in the lungs of Nlrp3^-/-^ mice than that of wildtype mice at 1 dpi, similar to wildtype mice at 3 dpi, and only somewhat less abundant at 6 dpi (Fig 5B). The Ly6G^hi^ mature neutrophils were lower in Asc^-/-^, Casp1/11^-/-^ or Aim2^-/-^ mice than that of Nlrp3^-/-^ mice at 1 dpi (S5 Fig). Further, numbers of detrimental pMDSC were also decreased in lungs of Nlrp3^-/-^ mice at 1 dpi (Fig 5C), whereas the number of these cells in Casp1/11^-/-^ and wildtype mice were similar. Numbers of F4/80^+^ macrophages were unchanged between Nlrp3^-/-^ and Casp1/11^-/-^ mice through day 3 post-infection, but declined by day 6 (Fig 5D). Immature and ineffective mMDSC were somewhat less abundant in the lungs of Nlrp3^-/-^ mice compared to Casp1/11^-/-^ and wildtype mice at 6 dpi (Fig 5E). Thus, the resistance of Nlrp3^-/-^ mice to lethal Ft infection is unlikely to result from improved numbers or function among mature macrophages or a reduction in immature, ineffective mMDSC, but instead correlates with increased numbers of mature neutrophils in the lung at 1 dpi.

The appearance of neutrophils in the lung on day 1 precedes reduced bacterial burden over the course of infection in Nlrp3^-/-^ mice, suggesting that Nlrp3 promotes host lethality in wildtype mice by restricting the appearance of these cells. Ft infected Nlrp3^-/-^ mice were therefore depleted of neutrophils with anti-Gr-1 antibody. Ft infected Nlrp3^-/-^ mice depleted of Gr-1^+^ cells succumb to infection (Fig 5F), indicating that the neutrophils observed at 1 dpi are critical for protection. We previously observed that lung recruited myeloid cells in the neutrophil lineage are mostly immature in Ft-infected mice [4]. As such, the immature neutrophils (pMDSC) and mMDSC population appear lower in Nlrp3^-/-^ mice, suggesting that eliciting mature neutrophils may be sufficient for protection. To further evaluate this idea, mice were administered a low dose of *E*. *coli* LPS (10 or 100 μg/mouse) by the intranasal route (Fig 5G). LPS-treatment elicited a mature neutrophil response with predominant neutrophils and some macrophages at 48 h post-treatment, but prior to infection (S5E Fig). Neutrophil numbers remained high in these mice at day 1 post-infection, but declined at later time points (Fig 5G), while the magnitude of the macrophage response, although higher at day 3, is similar to those seen in wildtype and Nlrp3^-/-^ mice infected with Ft LVS. As an alternative approach, we adoptively transferred neutrophils isolated from the bone marrow cells of the naïve wildtype mice. LPS stimulation prior to infection was completely protective and transfer of BM neutrophils protected approximately 30% of infected mice (Fig 5H). Further, Ft LVS was effectively controlled in the lungs of low-dose LPS treated mice (Fig 5I) and those receiving transferred neutrophils (Fig 5J). Thus, we conclude that Nlrp3 prevents lung recruitment, maturation, or survival of mature neutrophils, that are otherwise capable of promoting clearance of Ft which preserves lung architecture and increases the survival of mice during pulmonary infection.

The protective neutrophil response in Nlrp3^-/-^ mice may result from improved neutrophil recruitment. The cytokines/chemokines IL-17, mouse KC, and MCP-1 are important for myeloid cell recruitment and maturation [37]. We evaluated their expression in the lungs of wildtype and Nlrp3^-/-^ mice with Casp1/11^-/-^ mice as a control representing inflammasome-deficient mice. IL-17 was increased in Nlrp3^-/-^ mice at 1 dpi, was equivalent at 3 dpi, but reduced at 6 dpi compared to wildtype mice (Fig 5K), but levels of KC and MCP-1 did not differ between Ft infected Nlrp3^-/-^ and wildtype mice. Except for reduced IL-17 and slightly elevated KC levels at 6 dpi, the levels of these soluble mediators in the lungs of Ft infected Casp1/11^-/-^ mice were essentially identical to wildtype controls (Fig 5K). Since IL-17 levels increase concomitantly with the appearance of neutrophils in the lungs of Nlrp3^-/-^ mice at 1 dpi, IL-17 may be negatively regulated by Nlrp3 early during infection. In this case, neutralization of IL-17 is expected to reverse the protective phenotype leading to increased mortality. However, administration of anti-IL-17 did not alter the survival of Nlrp3-deficient mice (Fig 5L). Thus, although neutrophils are essential for protection against Ft in Nlrp3^-/-^ mice, IL-17 is dispensable during this protection. IL-1 also recruits neutrophils and IL-1r-deficient mice are susceptible to sublethal infection with Ft LVS. Accordingly, neutrophil recruitment to the lungs was significantly reduced in Ft LVS infected mice lacking the IL-1r (Fig 5M). As Nlrp3-deficient mice have reduced IL-1β production, the increased neutrophil numbers in the lung occur despite diminished IL-1β levels. These observations suggest that improved neutrophil recruitment may not account for their increased numbers and that other mechanisms should be considered.

### Nlrp3-mediated, inflammasome-independent, cell death contributes to lung pathology

**Fig 6:**
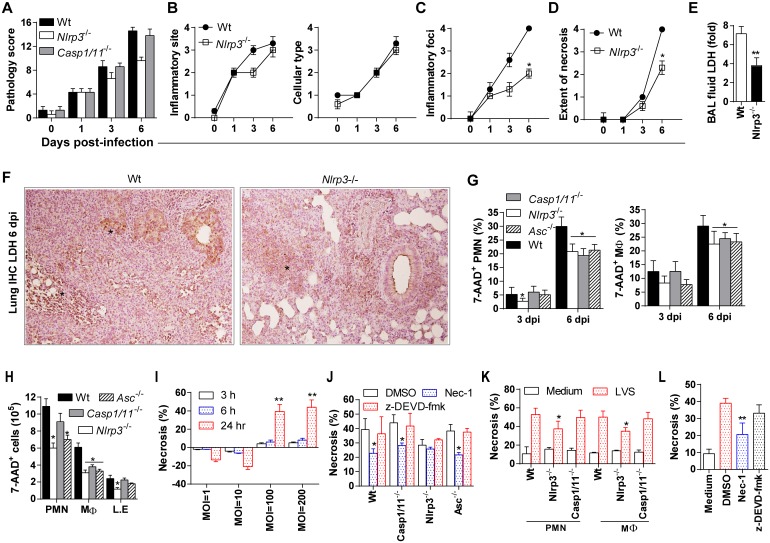
Tissue pathology and necrotic death of myeloid cells is reduced in Ft infected Nlrp3^-/-^ mice. (A) Lung pathology score for mice infected with lethal Ft LVS (1000 cfu). Pathology score was calculated by analysis of lung sections (n = 6 mice) for location/type/and extent of inflammation and necrosis (see Methods) (mean ± SD of two experiments, Mann-Whitney test, *p<0.05 indicates difference from wildtype mice). (B) Individualized pathology scores for inflammatory site and cellular types (mean ± SD of two experiments as shown in panel A). (C-D) Individualized pathology scores for number/size of inflammatory foci and extent of necrosis (mean ± SD of two experiments, n = 6 mice, Mann-Whitney test, *p<0.05). (E) LDH level in BAL fluid following LVS-infection (mean ± SD of two independent experiments, Student’s t-test, **p<0.01). (F) Positive immunoreaction for localization of LDH (asterisk), as an indicator of necrosis, in representative lung section at 6 dpi (IHC with hematoxylin counterstaining, 400x). (G) Per cent 7-AAD^+^ neutrophils (PMN) and macrophages (MØ) in Ft-infected lungs at 3 and 6 dpi (mean ± SD of two independent experiments, Student’s t-test, *p<0.05 indictaes difference from wildtype mice). (H) Total number of 7-AAD^+^ cells in Ft-infected lungs at 6 dpi (mean ± SD of two independent experiments, Student’s t-test, *p<0.05). (I) Per cent necrotic cells calculated by LDH release in BMDM infected with Ft LVS at increased MOI for different time points (mean ± SD of three independent experiments, Student’s t-test, **p<0.01 indicates difference from 3 and 6 h at specified MOI). (J) Per cent necrosis (LDH release) of BMDM pre-treated (30 min) with z-DEVD-fmk (50 μM) or Nec-1 (50 μM) and then infected with Ft LVS (MOI = 100) for 24 h (mean ± SD of three independent experiments, Student’s t-test, *p<0.05 indicates difference from LVS-infected cells treated with DMSO). (K) Per cent necrosis (LDH release) of PMN or lung macrophages infected with Ft LVS (MOI = 100) for 24 h (mean ± SD of two independent experiments, Student’s t-test, *p<0.05 indicates difference from wildtype cells). (L) Per cent necrosis (LDH release) of LA-4 cells infected with Ft LVS (MOI = 100) for 24 h (mean ± SD of three independent experiments, Student’s t-test, *p<0.05 indicates difference from LVS-infected cells treated with DMSO).

Our data suggest that Nlrp3 prevents an early neutrophil response that effectively controls Ft replication and potentially restricts overt cellular inflammation that contributes to tissue pathology and acute death. Consistent with the preservation of airway space (see Fig 2), overall lung pathology scores are lower in Nlrp3^-/-^ mice when compared to wildtype or Casp1/11^-/-^ mice (Fig 6A). However, a further analysis of individual criterion used for pathology scoring revealed that the site of inflammation (mostly at peri-bronchiolar, peri-vascular and alveolar regions) with involvement of neutrophil (PMN)/macrophages infiltration was essentially identical between wildtype and Nlrp3^-/-^ mice (Fig 6B). In contrast, Nlrp3^-/-^ mice had fewer inflammatory foci (mostly small and patchy) versus many large inflammatory foci seen in wildtype or Casp1/11^-/-^ mice (Fig 6C). Importantly, the extent of necrosis was also less severe in Nlrp3^-/-^ mice with fewer and smaller necrotic foci in the lung epithelial parenchyma (Fig 6D). Consistently, in the absence of Nlrp3, lung damage is significantly reduced as reflected by significantly lower LDH release in BAL fluid (Fig 6E) and *in situ* assessment of LDH release in lung tissue by immunohistochemistry (Fig 6F). Indeed, necrotizing inflammation is a hallmark of pulmonary tularemia and is associated with the death of myeloid cells which constitute the lethal pulmonary inflammatory response to Ft [4]. Next, we quantified the number of dead cells (7-AAD^+^) among the recoverable fraction of the single cell suspension obtained from lungs by flow cytometry *and in situ* histological evaluation of inflammatory/necrotic foci by microscopy. At day 3 post-infection, although limited, PMN death was evident and comparable between wildtype, Asc^-/-^, and Casp1/11^-/-^ mice, but reduced in Nlrp3^-/-^ mice as assessed by 7-AAD staining (Fig 6G). While macrophage death was also reduced in Nlrp3^-/-^ and Asc^-/-^ mice at day 3, death of macrophages from Casp1/11^-/-^ mice was comparable to wildtype. At day 6, approximately 30% of lung infiltrating PMN cells and macrophage from wildtype mice were dead. In contrast, death of these cells was reduced in Nlrp3^-/-^, Asc^-/-^, and Casp1/11^-/-^ mice. At day 6 when necrotic changes are most evident on histological observation, all inflammasome component-deficient strains had reduced numbers of dead PMN cells and macrophages (Fig 6H and S5 Fig). Consistent with lung necrosis scores (Fig 6D), only Nlrp3^-/-^ mice had significantly fewer dead lung epithelial cells. These results suggest that Nlrp3 drives the death of myeloid and epithelial cells and contributes to necrotic injury in the lung, yet various mechanisms including ASC and caspase-1-dependent processes also occur. However, unlike Nlrp3-deficient mice, ASC- and caspase-1/11-deficient mice show no protection against Ft LVS infection, no protective early neutrophil response, and no reduction in lung pathology. These observations are consistent with an inflammasome-independent role for Nlrp3 in promoting host mortality perhaps via Nlrp3-mediated myeloid cell death.

Nlrp3 is involved in two distinct forms of cell death, caspase-1 dependent pyroptosis [23, 38–42] and Asc-dependent, but caspase-1 independent pyronecrosis [23, 38, 43]. However, while apoptosis (caspase-3-mediated) and pyroptosis (caspase-1-mediated) have been implicated in Ft or Fn-induced macrophage death [5, 6, 14, 18, 29, 44], it is unclear whether Nlrp3 is involved. On *in vitro* infection, Ft induced necrosis in BMDM in a dose- and time-dependent manner at an MOI of 100 or greater at 24 hours post-infection (hpi) as measured by LDH release. At an MOI of 1 or 10, no cell death was observed up to 24 hpi (Fig 6I). As macrophages can also undergo necroptosis, a form of regulated/programmed necrotic cell death [45], we sought to distinguish between an apoptotic, pyroptotic, pyronecrotic, or necroptotic mechanism of Nlrp3-mediated cell death. While Ft-induced death of BMDM from Casp-1/11- and Asc-deficient mice was indistinguishable from wildtype controls, significantly less cell death was observed in Nlrp3^-/-^ BMDM (Fig 6J). Further, caspase-3 inhibitor (z-DEVD-fmk) treatment did not reduce Ft-induced cell death in BMDM from any of these mouse strains. This result suggests a novel Nlrp3-mediated form of cell death independent of caspase-3, caspase-1 and Asc. Interestingly, pre-treatment with necrostatin-1 (Nec-1), an inhibitor of RIP1/3-mediated necroptosis, reduced cell death in wildtype, *Casp1/11* ^-/-^, and Asc^-/-^ BMDM, but did not further reduce the death of Nlrp3^-/-^ BMDM. Further, necrosis was reduced in cultured alveolar macrophages (MØ) and PMN from Nlrp3^-/-^ mice, but was comparable for cells isolated from wildtype or Casp1/11^-/-^ mice (Fig 6K). Necrotic damage was also observed in lung epithelium in Ft-infected mice and reduced in the absence of Nlrp3. To examine whether Ft kills epithelial cells, we infected the LA-4 lung epithelial cell line with Ft LVS and examined LDH release at 24 hours. As with myeloid cells, death of these cells was inhibited by Nec-1, but was insensitive to caspase-3 inhibition (Fig 6L), suggesting that Ft elicits lung epithelial cell necrosis. Collectively, these data suggest that Nlrp3-dependent, but Asc, caspase-1/11, and caspase-3-independent cell death, which is likely necrosis/necroptotic, contributes to death of lung myeloid and epithelial cells during pulmonary tularemia.

As the deficiency of Nlrp3 limits neutrophil cell death early during infection and lung epithelial cell death later in infection, Nlrp3-dependent necroptosis might help explain the pathology and host mortality accompanying pulmonary tularemia. Ft-induced necrosis is blunted in myeloid cells from Nlrp3-/- mice and Nec-1 protects cultured cells for Ft-elicited cell death, but the behavior of mature cells *in vitro* may not reflect the mechanism contributing to tissue damage and death.

### Necrostatin recapitulates the survival phenotype of Nlrp3-deficient mice

**Fig 7:**
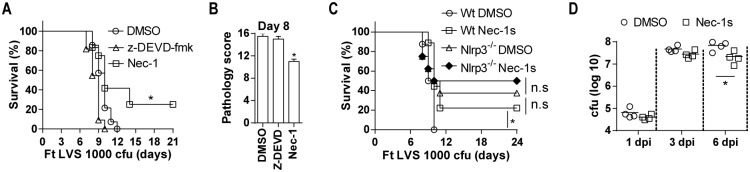
Necrostatin-1s treatment protects Ft infected mice. (A) Survival of wildtype mice infected with Ft LVS and then treated i.p. with z-DEVD-fmk (200 μg/mouse) or Nec-1 (200 μg/mouse) daily between 2–6 dpi (%survival of two independent experiments, n = 10 mice, Log rank test). (B) Lung pathology scores wildtype mice infected with Ft LVS and then treated i.p. with z-DEVD-fmk (200 μg/mouse) or Nec-1 (200 μg/mouse) as above (mean ± SD of two experiments, n = 4 mice, Student’s t-test, *p<0.05. (C) Survival of wildtype and Nlrp3^-/-^ mice infected with Ft LVS and then treated i.p. with and without Nec-1s (200 μg/mouse) daily between 2–6 dpi (% survival of two independent experiments, n = 10 mice, Log rank test, n.s. = p>0.5). (D). Lung bacterial burden in wildtype mice infected with Ft LVS and then treated i.p. with Nec-1s as above (mean ± SD of four mice, Student’s t-test**p<0.01).

Following lethal LVS infection, 25% of mice treated with Nec-1 survived (Fig 7A) and necrotic lung damage was also reduced (Fig 7B), while wildtype control mice and those treated with a caspase-3 inhibitor were not protected and had comparable pathology. As Casp1/11-/- mice also showed no protection (Fig 3), these data suggest that Nec-1 sensitive necroptotic cell death contributes to host mortality and that caspase-1- or caspase-3-dependent forms of cell death do not. During the course of these studies, we learned that in addition to RIPK1, Nec-1 also inhibits inhibits IDO [46], which could complicate our results. However, the Nec-1 derivative Nec-1s retains RIP1K specificity but does not inhibit IDO [46]. Therefore we repeated our survival experiments using Nec-1s. As with Nec-1 treatment, approximately 25% of mice treated with Nec-1s survived lethal Ft LVS infection (Fig 7C). Importantly, Nec-1s treatment of Nlrp3-deficient mice did not significantly improve the resistance of these mice to lethal Ft infection (Fig 7C), strongly suggesting that Nlrp3 and RIPK1 are acting on the same pathway. Further, Nec-1s treatment of wildtype mice resulted in improved bacterial control (Fig 7D), similar to that seen with Ft LVS infected Nlrp3-deficient mice. These data support the hypothesis that Nlrp3 mediates a necroptotic program that contributes to irreversible damage of the lung during lethal pulmonary tularemia. Overall, our findings support the conclusion that during pulmonary tularemia Nlrp3 drives host mortality in an inflammasome-independent fashion that prevents an early neutrophil response important for host protection by promoting myeloid cell death. Moreover, the pathologic effects of Nlrp3 can be inhibited by the RIP1 inhibitor, Nec-1s, strongly implicating Nlrp3-dependent cell death as a key determinant of host susceptibility to Francisella.

## Discussion

Pulmonary tularemia is a frequently fatal, acute necrotic pneumonia in humans and animals caused by various sub-species of the environmental bacterium *Francisella tularensis* (Ft). Most human cases of pulmonary tularemia result from infection with *F*. *tularensis holarctica* (the parent strain of the live vaccine strain; LVS) or *F*. *tularensis tularensis* (e.g. SchuS4), while and *Francisella novicida* (Fn) cause disease in rodents. As a vaccine strain, Ft LVS is non-pathogenic to humans and Fn rarely causes human disease [1–3]. Despite intensive research efforts, the mechanism by which Ft elicits fatal disease is poorly understood. Many studies have reported that the Asc/Caspase-1 axis and, in particular, the Aim2, inflammasome which generates IL-1β and IL-18 is critical for resistance to Fn [14–19]. Owing to the emphasis on IL-1β and the still unexplained inability of Fn to elicit an Nlrp3 inflammasome response in mouse macrophages, the role of Nlrp3 during Ft infection has not been explored further. In addition, with the exception of a few studies [13, 28], the roles played by Nlrp3, Aim2 or other inflammasomes during infection with Ft LVS and SchuS4 have not been investigated.

Given the extensive genetic similarity (97% nucleotide identity) between Ft and Fn [47], differences in virulence and pathogenesis are thought to arise from differential regulation of homologous genes and distinct roles for their products [3]. We have noted that both Fn and Ft LVS are capable of utilizing the NLRP3 inflammasome in human cells [22]. Thus, the present study sought to examine the role of Nlrp3 in the mouse model of pulmonary tularemia caused by Ft LVS or SchuS4. Interestingly, despite Ft strain-specific differences in the IL-1β/IL-18 responses of bone marrow-derived macrophages and differential reliance upon Nlrp3 or Aim2 inflammasomes, Nlrp3-deficient mice exhibited various degrees of resistance (decreased susceptibility) to lethal pulmonary infection with Fn, Ft LVS, and SchuS4. This finding demonstrates, for the first time, that Nlrp3 is activated by *Francisella* species in general, acting as a host susceptibility factor driving the pathogenesis of pulmonary tularemia. Consistent with improved resistance, lung bacterial burden and lung pathology were significantly reduced in Ft-infected Nlrp3^-/-^ mice. Mice deficient for Caspase-1/11, Aim2, or Asc, however, displayed increased susceptibility despite levels of inflammatory cytokines similar to Nlrp3-deficient mice, clearly indicating an inflammasome-independent role for Nlrp3 in the pathogenesis of pulmonary tularemia. Our results confirm that Asc and Caspase-1 contribute to protection, while Nlrp3, independent of inflammasome, is detrimental during Ft infection [14–19, 38–43]. We find that Nlrp3-deficient mice exhibit an early, protective, mature neutrophil response accompanied by reduced numbers of immature myeloid cells, a response that is absent in wildtype mice and mice lacking Caspase-1. Our results suggest that Nlrp3 prevents this mature response, perhaps via Nlrp3-dependent cell death, and that Nlrp3 contributes to a dysregulated myeloid cell response that drives necrotic pathology and host susceptibility during pulmonary tularemia.

Prior to this study, the role of inflammsome activating proteins important for IL-1β/IL-18 responses in mice following infection with Ft strains other than Fn was essentially unknown. Unlike Fn which relies almost exclusively upon Aim2 inflammasome for macrophage production of both IL-1β and IL-18, Ft LVS and SchuS4 utilize both Aim2 and Nlrp3. However, we noted an interesting difference between Ft LVS and SchuS4 in their activation of Aim2 and Nlrp3 in macrophages. While the IL-1β response to SchuS4 strictly required Nlrp3, Aim2 was only partially required. However, Ft LVS required Nlrp3 for IL-1β responses, which were approximately 2-fold higher in the absence of Aim2. IL-18 responses to either Ft LVS or SchuS4, however, were dependent upon both proteins. Curiously, Aim2-independent production of IL-1β following Ft LVS infection was not observed *in vivo*. This disparity suggests that aspects of inflammasome activation in macrophages may differ based on *in vivo* versus *in vitro* context or may reflect the phenotypic response of mature macrophages *versus* the immature cells found in the lungs of Ft infected mice [4]. Despite these differences, how the Nlrp3-inflammasome is engaged in Ft infection and how Fn relies entirely upon the Aim2 inflammasome in macrophages remain unknown. Consistently both Aim2 and Nlrp3 require the Asc adapter protein [20, 48], suggesting the possibility of negative regulation via competition for Asc. Fn replicates faster intracellularly and causes macrophage cell death more rapidly than Ft LVS. It is possible that release of bacterial and cellular dsDNA following cell demise could engage the Aim2-inflammasome. Interestingly, with Ft LVS macrophages, Aim2 seems to negatively regulate the Nlrp3 response, as Aim2^-/-^ cells generate higher amounts of IL-1β. Although we do not further pursue the mechanism responsible for increased IL-1β release in Aim2^-/-^ mice following Ft LVS infection in this report, this effect was consistently observed in all our experiments.

The increased susceptibility of Casp1/11^-/-^, Asc^-/-^, and IL-1r1^-/-^ mice to Ft LVS infection in this study and IL-1β-/- mice in a previous study [13] clearly underscore the critical requirement of IL-1β/IL-18 for protective immunity. Consistently, Casp1/11^-/-^ and Asc^-/-^ mice had reduced level of processed IL-1β/IL-18. In contrast, while Nlrp3^-/-^ and Aim2^-/-^ mice had levels of IL-1β and 1L-18 similar to those of Caspase-1/11 and Asc-deficient mice; only mice lacking Nlrp3 were protected from lethal Ft infection. Further, the Nlrp3 inflammasome inhibitor MCC950 did not result in any protection. This clearly indicates an inflammasome and IL-1/IL-18-independent mechanism for Nlrp3 that promotes susceptibility. As IL-1β/IL-18 levels are reduced in Ft LVS infected Nlrp3^-/-^ mice, the amounts of these cytokines required for protection during pulmonary tularemia may be much lower than previously thought. Alternatively, higher levels of IL-1β or IL-18 in the absence of Nlrp3 might afford greater protection. Consistent with this alternative, IL-1r1 is thought to be important for the generation of protective Ft-LPS specific IgM [13], however we observed no significant alterations in anti-Ft-LPS IgM in inflammasome component-deficient mice. Moreover, MCC950 treatment increased the susceptibility of wildtype mice receiving a 50% lethal dose of Ft LVS, presumably due to reduced IL-1β/IL-18 production. Thus, the precise role that IL-1β and IL-18 play in the absence of Nlrp3 as well as the levels required for protection remain to be addressed.

Infiltration of the Ft-infected lung by immature myeloid cells/myeloid-derived suppressor cells (MDSC) is associated with necrotic lung damage and host death, as we recently reported [4]. Appearance of these immature cells was comparable between wildtype controls and Capase-1/11-deficient mice lacking intact Nlrp3 and Aim2 inflammasome function. Indeed, Caspase-1/11- and Aim2-deficiency is associated with ineffective clearance of Ft, as evidenced by bacterial burdens comparable or higher than control mice later in infection. Thus, the immature myeloid response appears to be inflammasome-independent. However, improved clearance of Ft in Nlrp3-deficient mice correlates with a necessary early (day 1) mature neutrophil (PMN) response that appears critical for inhibiting further replication of Ft in the lungs and a corresponding decrease in PMN-MDSC at 6 dpi. This is consistent with our recent demonstration that a mature neutrophil response in sub-lethal Ft LVS infection supports bacterial clearance and survival [4] and with the protection against Ft LVS infection provided by eliciting mature neutrophils with low dose intranasal instillation of LPS or by direct transfer of neutrophils. Immature monocytic cells (mMDSC) declined late during Ft LVS infection of Nlrp3^-/-^ mice, but at the same time point, mature F4/80+ macrophages were also decreased. Yet, although similar in magnitude, macrophage numbers were higher with low-dose LPS instillation. In contrast to the clear role of neutrophils, whether mature macrophages or reduced numbers of mMDSC also contribute to bacterial control and resistance in these mice is unknown.

Recently, it has been reported that Nlrp3 regulates chemokine-mediated functions and recruitment of neutrophils contributing to hepatic ischemia-perfusion injury independent of inflammasome [43]. In this previous study, Nlrp3 regulates the function of KC and thereby reduced neutrophil recruitment in Nlrp3^-/-^ mice when compared to Asc^-/-^ or Casp1^-/-^ mice. However, excluding a significant, but seemingly unnecessary increase in IL-17 levels at day 1 post-infection in Nlrp3-/- mice, differences in the level of KC or MCP-1 that might impact myeloid cell recruitment were not discernable between Nlrp3^-/-^, Asc^-/-^, Casp1/11^-/-^ and Aim2^-/-^ mice. This lack of difference suggests that the early, protective, neutrophil response is independent of the actions of these chemokines as well as IL-17. This is somewhat surprising as IL-17 has been implicated in promoting Th17 responses that appear to be protective in mice infected with an Ft LVS mutant which fails to elicit PGE_2_ [49]. IL-1 is important for neutrophil recruitment during Ft LVS infection, but Nlrp3-deficient mice have increased neutrophil numbers despite diminished IL-1β. These early neutrophils may represent the lung-associated, marginated pool of neutrophils which may be mobilized to the lung upon infection, but how this might occur during infection has not been evaluated [50–51]. Whether Nlrp3 directly or indirectly negatively regulates neutrophil maturation or recruitment is unclear. Alternatively, death of neutrophils owing to Nlrp3 may be responsible for restricting the number of mature neutrophils.

Acute necrotic lung injury correlates with dying myeloid cells, loss of pulmonary function, and death during lethal Ft infection [4]. Consistent with sustained recruitment of immature myeloid cells/MDSC, Casp1/11^-/-^ mice exhibited severe necrotizing inflammatory changes in the lung. However, the size and number of inflammatory foci in the lung and accompanying necrotic damage was markedly lower in Nlrp3^-/-^ mice resulting in notable preservation of lung architecture. These changes point to an Nlrp3-mediated cell death mechanism. Curiously, preservation of lung architecture has been observed previously in Nlrp3-deficient mice infected with Klebsiella, but these mice nevertheless succumb due to insufficient IL-1β production [39].

Nlrp3 is reported to induce two forms of cell death. The first is inflammasome-dependent pyroptosis and requires caspase-1 activation [23, 38–42]. The second is Nlrp3- and Asc-dependent, but Casp-1-independent and termed pyronecrosis [39, 43]. Importantly, both share some features of classical necrosis and are appreciated as distinct forms of programmed cell death [23]. It is unclear which forms of cell death mediate the necrotic damage evident during lethal pulmonary tularemia. Intriguingly, severe necrosis during pulmonary tularemia requires Nlrp3, but appears largely independent of caspase-1 and Asc in both myeloid and lung epithelial cells. Our *in vitro* infection data suggest that Ft-induced BMDM cell death is non-apoptotic (Caspase-3 independent), non-pyroptotic (Caspase-1-independent), and non-pyronecrotic (Asc-independent). Our data also suggest that an Nlrp3-dependent necrosis/necroptotic (Nec-1-sensitive) pathway likely predominates in myeloid cells dying during Ft infection. Although Nlrp3 function in myeloid-lineage cells is well documented, Nlrp3 expression in epithelial cells also plays a critical role during inflammation [52–56]. We also observe reduced lung stromal cell death in Ft infected Nlrp3-deficient mice and Ft-infected epithelial cells die by a mechanism that is also sensitive to inhibition with Nec-1. While *Francisella* has been reported to induce caspase-3 activation and apoptotic cell death in the lung [5, 6], our data suggests that caspase-3 may be less important by implicating Nlrp3 in a form of cell death distinct from apoptosis, pyroptosis, and pyronecrosis. Indeed, Ft LVS infected mice treated with a caspase-3 inhibitor are not protected, while treatment with the RIPK1 inhibitor Nec-1s is protective and results in reduced bacterial burden. Whether RIPK1 is responsible for restricting the early mature neutrophil response by promoting the death of these cells is unclear. Nec-1s treatment does not result in an increase in lung neutrophils following Ft LVS infection, while macrophage numbers are higher at days 3 and 6 post-infection (S7 Fig), suggesting that the early neutrophil response is restricted by Nlrp3, but not by RIPK1. However, DMSO is known to diminish lung neutrophil numbers [57] and reduces both neutrophil and macrophage bacteriocidal function without enhancing host susceptibility to infection [58], important caveats suggesting that differences in neutrophil survival may be obscured. Such inhibition may also account for the larger bacterial numbers and less pronounced bacterial control seen in the Nec-1s experiment. Of note, Nec-1s treatment does not significantly improve the survival of Ft LVS infected Nlrp3-deficient mice, suggesting that Nlrp3 activation of RIPK1, whether direct or indirect, is likely. This supports the hypothesis that Nlrp3 promotes lethality through inducing necroptosis, although the cell populations critically impacted *in vivo* and how necroptosis contributes to the lack of an early, protective neutrophil response are still unknown. In addition, the precise mechanisms of cell death involved during infection remain unclear as at later times post infection, myeloid cell death also appears to involve Asc and Caspase-1/11. Deciphering whether Nlrp3 is required for the apoptotic cell death observed during infection with the Type A strain SchuS4 [5, 6], a caspase-3 independent mechanism, or both, will be of considerable interest and require further study. More importantly, dissecting how Nlrp3 is involved in epithelial cell death and whether dying myeloid cells or direct infection are responsible for necrotic damage to the lung stroma will likely be of interest to those interested in the pathogenesis of acute lung injury during acute necrotic pneumonias and others exploring the functions of Nlrp3. Lastly, early infiltration of neutrophils is important for protection and likely controls Ft numbers, which may ultimately reduce myeloid and epithelial cell death. These cells may represent the pulmonary-associated marginated pool of neutrophils that are thought to be poised to respond to infection. If demonstrated, susceptibly of these to Nlrp3-mediated cell death would isolate a role for Nlrp3 in restricting a key step in the appearance of neutrophils early during pulmonary infection.

Are there other inflammasome-independent mechanism involving Nlrp3 that may contribute to our observations? Recently, Nlrp3 was demonstrated to cooperate with IRF4 that drives IL-4 transcription and positively regulate differentiation of Th2 cells [59]. In this system, Nlrp3 deficiency increased Th1-dependent responses which exerted significant control of disease in mouse models of asthma and metastatic melanoma [59]. In contrast, during Leishmaniasis, Nlrp3 also promotes Th2-biased adaptive immunity in an inflammasome-dependent manner through IL-18 [60]. Although these two studies report divergent mechanisms by which Nlrp3 favors Th2 immune responses, it is understood that Nlrp3 plays a critical role in restricting Th1 responses. However, in the Ft infection model we observed no decrease in Th2 cytokines such as IL-4 or IL-10 in Nlrp3-deficient mice at any time following infection, but IFNγ production was slightly elevated at 6 days post-infection (S3F Fig). Importantly, lung necrosis is already moderated at 3 days post-infection in Nlrp3^-/-^ mice, at which point bacterial numbers in the lung also appear to be declining. Given the requirement for the mature neutrophils early in infection and the late appearance of elevated IFNγ, it is unlikely that resistance to Ft in the absence of Nlrp3 results from transcriptional alterations in lung Th1/Th2 cytokines during pulmonary tularemia.

Collectively our data demonstrates that Nlrp3 acts as a host susceptibility factor during Francisella infection. Independent of its role in the inflammasome, Nlrp3 prevents the appearance of neutrophils in the lung early during Francisella infection and ultimately contributes to lung damage and host mortality likely via necrotic/necroptotic death of myeloid and stromal cells.

## Materials and Methods

### Mice

C57BL/6J wild-type, Aim2^-/-^ (Aim2^Gt(CSG445)Byg^), *Casp1/11* ^-/-^ (Casp1^tm1Flv^), IL-1r-/- (Il1r1^tm1Imx^), and CD45.1 (B6.SJL-Ptprc^a^ Pepc^b^/BoyJ) congenic mice were purchased from Jackson laboratories. Nlrp3^-/-^ and Asc^-/-^ mice were described previously [61]. All the mice were housed and bred in the Animal Resources Facility at Albany Medical College. Experiments were conducted using male and female mice (8–10 weeks).

### Ethics statement

All animals were maintained in the animal resource facility at Albany Medical College and handled in strict accordance with good animal practice as defined by the United States Public Health Service and Department of Agriculture. All animal work was approved (ACUP #12–04001, 12–04002 and 12–04003) by the Albany Medical College Institutional Animal Care and Use Committee (IACUC) and adhered to the regulations of the Public Health Service (PHS) policy on Humane Care and Use of Laboratory Animals.

### Intranasal Ft infection

Ft SchuS4 and LVS were cultured in modified Muller Hinton (MH) or Brain Heart Infusion (BHI) broth as described [62]. All experiments utilizing SchuS4 were conducted within the Albany Medical College, CDC-certified BSL-3 facility. Bacterial inocula were prepared in sterile PBS by serial dilution to defined cfu numbers. Mice were anesthetized by i.p injection of 80–100μl/mouse of Ketamine (20mg/ml) and Xylazine (1mg/ml) mixture. Anesthetized mice were infected i.n with 40 μl of inoculum instilled in a single nare. An equal volume of inoculum was plated on MH chocolate agar to confirm actual cfu numbers. Sham-inoculated controls received an equal volume of PBS or appropriate vehicle medium.

### Necropsy, tissue collection, histology and immunohistochemistry

Blood was collected by submandibular venipuncture [63] and mice were euthanized with a mixture of Ketamine and Xylazine followed by cervical dislocation. Necropsy was performed, gross lesions were noted, and organs (lungs, liver and spleen) were collected aseptically to prepare tissue homogenate (for bacterial counting and/or cytokine measurements), single cell suspensions (for immunophenotyping), or histology (for pathological assessment) as described previously [4]. For lung homogenate preparation, either whole lungs or a half of the lungs containing pieces (consistent size for each mouse) from middle lobe, post-caval lobe, the right superior lobe and the left lung lobes were collected in sterile PBS. For histology, either the entire lung lobes or representative pieces each from the right superior and inferior lobes and a half of the left lung lobes were collected in 10% buffered formalin. Either all or half of the spleen was collected in formalin for histology. As well, pieces of liver from left lateral lobe and medial lobe were collected in formalin. Formalin fixed tissues were processed by standard histological procedures and 4μm-thick sections were cut and stained with hematoxylin and eosin (HE). Sections of lungs, spleen or liver were examined for the location of inflammatory foci, type of infiltrating cells and the extent of necrotic changes in parallel with sections from uninfected or Ft-infected lungs and scored using the criteria described previously [4]. Immunohistochemical (IHC) analysis for identification of myeloid cell types (Ly6G+, Ly6C+, or CD11b+) or localization of LDH was performed in formalin-fixed paraffin-embedded tissue sections, as described previously [4].

### Bronchoalveolar lavage (BAL) fluid collection

BAL fluid was collected from control and Ft-infected mice as described previously [4]. The cell-free clear supernatants were used immediately for LDH assays or stored frozen at -80°C for protein estimation. LDH assay was performed following manufacturer’s instruction using the Cytotox96 non-radioactive kit (Promega). The cell pellets were used for flow cytometry or cytospin smear preparation for differential cell counting (Giemsa).

### Bacterial burden estimation

Tissue homogenates prepared from whole or pieces of lungs/spleen/or liver were plated onto MH chocolate agar as described previously [4]. After 2 days, colony counts were performed and bacterial numbers (cfu) were calculated. Results are expressed as log_10_ cfu/ml/organ.

### Cytokine/chemokine/eicanosoids measurement

By using Mouse Group I and II Luminex assay kits (BioRad), cytokines/chemokines were estimated in clear tissue homogenates [4].

### Immunophenotyping by flow cytometry

Myeloid and lymphoid cell types were evaluated by multi-color flow cytometry as described previously [4]. Briefly, single cell suspensions from collagenase-digested lung or spleen were surface stained with either lymphoid markers lymphoid markers (CD3, CD4, CD8, NK1.1, B220, CD19, Terr119), myeloid markers (CD11b, CD11c, F4/80, Gr-1, Ly6C, Ly6G) and/or cell activation markers (CD80, CD86, MHCII, PD-L1 or CD115) for 30 min. Cells were fixed in 1% paraformaldehyde (PFA) and cytometry was performed on an LSRII (Becton Dickinson). For cell death analysis in lung cells, surface marker stained cells were stained with 7-AAD, washed twice and fixed in 1% PFA, prior to run in LSRII. Flow cytometry data were analyzed using FlowJo software (v10.0.1). Specific cell populations are represented as a mean percentage or total numbers for Ft-infected mice at various dpi in comparison to uninfected control mice (0 dpi).

### Isolation and culture of mouse macrophages

Bone marrow cells were isolated from the femurs and tibias of six to eight week-old mice to enrich bone marrow-derived macrophages (BMDM) as described previously [58]. F4/80+ lung macrophages were isolated from BAL fluid and Gr-1+ PMN cells were isolated from bone marrow cells by using magnetic beads (Miltenyi) as described previously [4]. Cells were cultured in DMEM containing L-cell supernatant and adherent cells were used for the infection studies.

### *In vitro* infection of mouse macrophages

Cells were infected with Ft LVS at different MOI (1, 10, 100 or 200) and cell death analyses were done at different time points (3, 6 or 24 h). In some experiments, cells were pre-treated (30 min) with z-DEVD-fmk (50 μM, final concentration), Nec-1 (50 μM) or vehicle alone and infected with LVS at MOI = 100. After 24 h, cell culture supernatants were tested for LDH activity as described [4].

### Cell death analysis

For lung cells, single cell suspensions were stained with surface markers followed by 7-AAD staining and analyzed in LSRII as described (P). Within myeloid cell subsets, the frequencies of 7-AAD+ cells were identified. For *in vitro* cultured cells, medium was removed; cells were harvested gently and stained with 7-AAD or TUNEL kit, or both, and then analyzed in an LSRII to count dead cells. BAL fluid and cell culture supernatants were tested for LDH activity as described [4].

### Adoptive transfer of PMN, eliciting myeloid cell response, and LVS infection

Mature Gr-1^+^ PMN cells were isolated from the bone marrow cells of CD45.1 donor mice and the cells (1 x 10^6^) were transferred to a group of naïve recipient CD45.2 mice by intra-tracheal intubation as described previously [4]. As well, naïve CD3^+^ T cells isolated from spleen were transferred to other group of recipient mice. The next day, these mice were infected with Ft LVS (1000 cfu) and survival was monitored. In other experiments, to elicit mature myeloid cell responses, naïve C57BL/6 mice were instilled i.n with LPS (*E*. *coli*, O55:B5) at 100μg or 10μg/mouse. After 48 h, these mice were infected with Ft LVS (1000 cfu) and survival was monitored. Two mice in each group were euthanized at 48 hr to analyze the BAL fluid cell counts and were found to have more number of cells than naïve control mice.

### Depletion of Gr-1^+^ PMN

For depletion of PMN cells, mice were i.p injected with anti-Gr-1 (RB8-8C5, BioXcell) or isotype control rat IgG2b mAb antibody (200μg/mouse) at 1 day prior and after Ft LVS infection. Cell depletion antibodies were purchased from BioXcell (Lebanon, NH). Antibody depleted mice were infected with Ft LVS (1000 cfu) and survival was monitored.

### Cytokine neutralization

Mice were administered i.p with anti-IL-17 antibody (Rat IgG1, clone TC11-18H10.1; Biolegend) or its isotype control antibody (200μg/mouse) at 1 and 3 dpi. Following infection, these mice were monitored for survival.

### Treatment of mice with selective inhibitors

At indicated experiments, Ft-infected mice were treated i.p. with Casp3-inhibitor z-DEVD-fmk (200 μg/mouse), Nec-1 (200 μg/mouse) or Nec-1s (200 μg/mouse) daily between 2–6 dpi. Ft-infected mice were treated with MCC950 (250μg/mouse) or glyburide (500μg/mouse) daily between 2–7 dpi and monitored for survival.

### Statistical analysis

Statistical analysis and data compilation were done using GraphPad Prizm (ver 6). Student’s t-test or a parametric ANOVA test with Tukey’s post-test was used for statistical comparisons between groups. For survival analysis, Log-rank (Mantel-Cox) test was used. The p<0.05 was considered significant.

## Supporting Information

S1 Fig (related to Fig 1) *F*. *tularensis* and *F*. *novicida* activate both Nlrp3- and Aim2-inflamamsome. (A) Levels of IL-12p40, TNF, MCP-1 and IL-10 measured in culture supernatants of wildtype and Nlrp3^-/-^ BMDM infected with Ft LVS at MOI = 100 for 24 h (mean ± SD of three independent experiments, Student’s t-test). (B) Intracellular bacterial burden after 4 h infection (mean ± SD of two experiments). (C) Western blot for proIL-1β in cell lysate of BMDM infected with Ft LVS. (D) Per cent cell death (LDH release) in BMDM infected with Ft LVS or F. novicida (MOI = 100) for 24 h (mean ± SD of two independent experiments, Student’s t-test, *p<0.05 indicates difference from wildtype cells).
(TIF)

S2 Fig (related to Fig 2). Nlrp3^-/-^ mice are less susceptible to lethal pulmonary Ft infection. (A) Bacterial burden in spleen and liver following Ft LVS infection (mean ± SD of three independent experiments, n = 9, Student’s t-test). (B) Survival of mice following 4 LD_100_ and 20 LD_100_ Ft LVS (1000 cfu) infection (% survival of two independent experiments, n = 12, Log-rank (Mantel-Cox) test, *p<0.05) and. (C) Per cent body weight loss following Ft LVS infection (mean ± SD of three independent experiments). (D) Survival of mice following Ft SchuS4 (150 cfu) infection (% survival of two independent experiments, Log-rank test).
(TIF)

S3 Fig (related to Fig 3). Nlrp3 mediates pathogenesis of pulmonary tularemia in an inflammasome-independent manner. (A) Levels of IL-1β measured in lung homogenates at indicated days post-Ft SchuS4 infection (mean ± SD of three mice, Student’s t-test, **p<0.01 indicates difference from wildtype mice). (B) Levels of IL-6 and TNF measured in lung homogenates at indicated days post-Ft SchuS4 infection (mean ± SD of three mice, n = 6, Student’s t-test) (C) Survival of Ft SchuS4 (10 cfu) infected mice (% survival of two independent experiments, n = 10, Log-rank test). (D) Bacterial burden in spleen and liver following Ft LVS infection (mean ± SD of two independent experiments, n = 6, Student’s t-test). (E) Serum cytokine levels in LPS-injected (10 mg/kg bwt) mice treated with MCC950 (1 mg/mouse (50mg/kg bwt) or 0.25mg/mouse (12.5mg/kg bwt) daily at 2–7 dpi) (mean ± SD of three mice, Student’s t-test, **p<0.01). (F) Levels of IL-4, IL-10, and IFNɣ in lung homogenates at indicated days post Ft LVS infection (mean ± SD of 6 mice).
(TIF)

S4 Fig (related to Fig 4) Innate antibody response is dispensable for protection during acute phase of tularemia. (A) Levels of anti-Ft IgG and IgA antibodies measured in BAL fluid of wildtype mice infected with Ft LVS (mean ± SD of OD_450_ from two independent experiments, n = 6, Student’s t-test).
(TIF)

S5 Fig (related to Fig 5). An initial neutrophil response in Nlrp3^-/-^ mice is protective during pulmonary tularemia. (A) Total numbers of Ly6G^+^ neutrophils in LVS-infected lungs (mean ± SD of two independent experiments). (B) Total numbers of cells recovered from LVS-infected lungs (mean ± SD of two independent experiments, n = 6, Student’s t-test). (C) Frequency of CD11b^+^ myeloid cells, Ly6G^+^ neutrophils, and F4/80^+^macrophages in LVS-infected lungs (mean ± SD of two independent experiments, n = 6, Student’s t-test. (D) Total numbers of CD3^+^ T cells and NK1.1+ cells in LVS-infected lungs (mean ± SD of two independent experiments, n = 6, Student’s t-test. (E) Total numbers of PMN and MØ in LPS-treated lungs after 48 hours without Ft LVS infection (mean ± SD of three mice, Student’s t-test, *p<0.05, **p<0.01).
(TIF)

S6 Fig (related to Fig 6). Nlrp3^-/-^ mice had less dead cells in lungs. (A) Histological sections of lungs from LVS-infected mice at 6 dpi show inflammatory foci, massive necrosis and clumps of dead cells (arrow). Note abundant dead cells with degenerated nuclei as clumps (arrow) in lung sections from wildtype, Casp1/11^-/-^ and Aim2^-/-^ mice, while moderate numbers in Asc^-/-^ mice and less number in Nlrp3^-/-^ mice (HE, 400x).
(TIF)

S7 Fig (related to Fig 7). Necrostatin-1s treatment protects Ft infected mice. Total numbers of PMN and MØ in LVS-infected mice treated with DMSO or Nec-1s (mean ± SD of three mice, Student’s t-test, *p<0.05, **p<0.01).
(TIF)
