# Supplementary figures and images for: Inflammasome-Independent NLRP3 Restriction of a Protective Early Neutrophil Response to Pulmonary Tularemia
Source: PLoS Pathog. 2016 Dec 7;12(12):e1006059. doi: 10.1371/journal.ppat.1006059 (PMC5142794; doi:10.1371/journal.ppat.1006059)

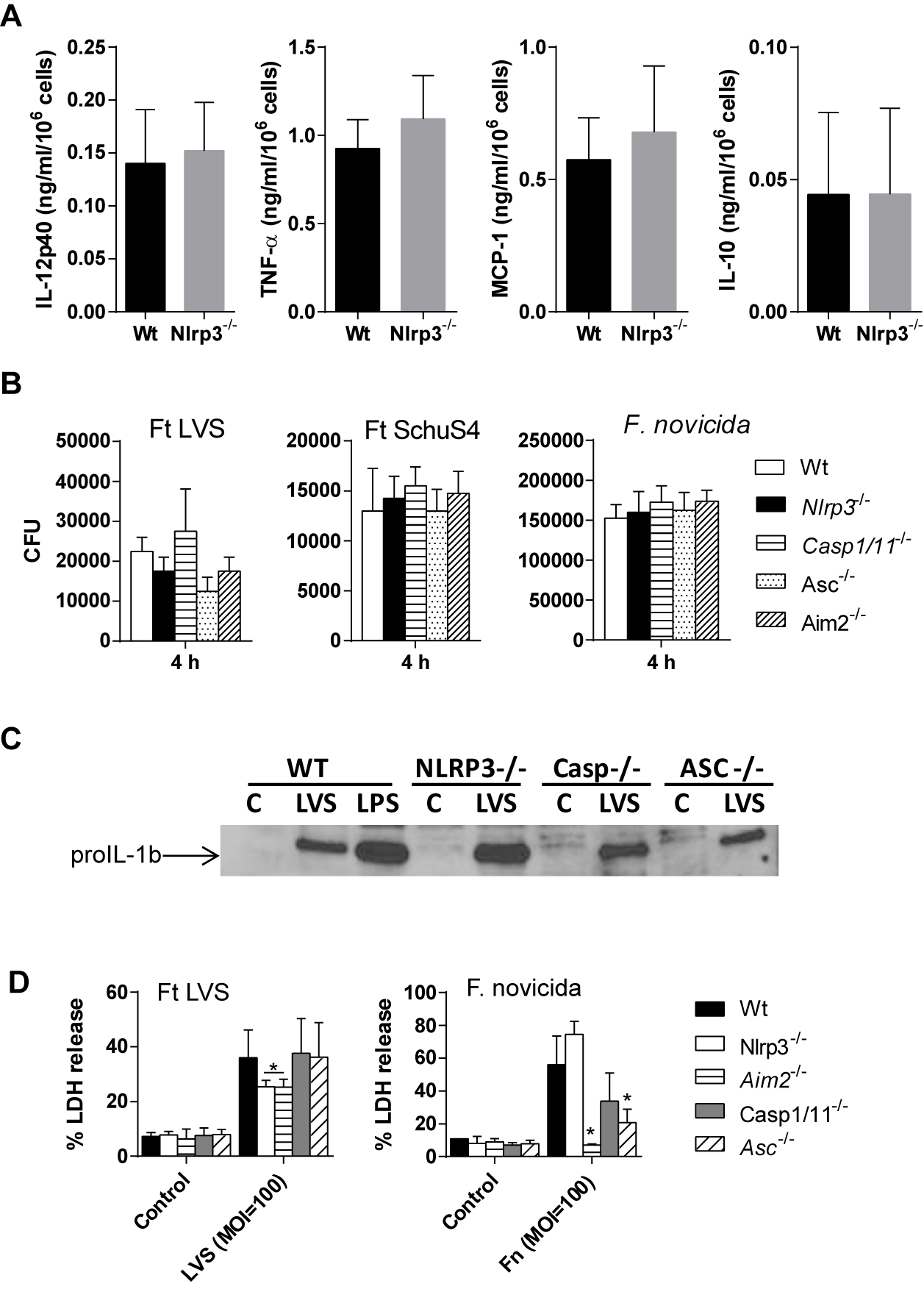

Supplement: S1 Fig — (A) Levels of IL-12p40, TNF, MCP-1 and IL-10 measured in culture supernatants of wildtype and Nlrp3-/- BMDM infected with Ft LVS at MOI = 100 for 24 h (mean ± SD of three independent experiments, Student’s t-test). (B) Intracellular bacterial burden after 4 h infection (mean ± SD of two experiments). (C) Western blot for proIL-1β in cell lysate of BMDM infected with Ft LVS. (D) Per cent cell death (LDH release) in BMDM infected with Ft LVS or F. novicida (MOI = 100) for 24 h (mean ± SD of two independent experiments, Student’s t-test, *p<0.05 indicates difference from wildtype cells). (TIF) [file ppat.1006059.s001.tif]

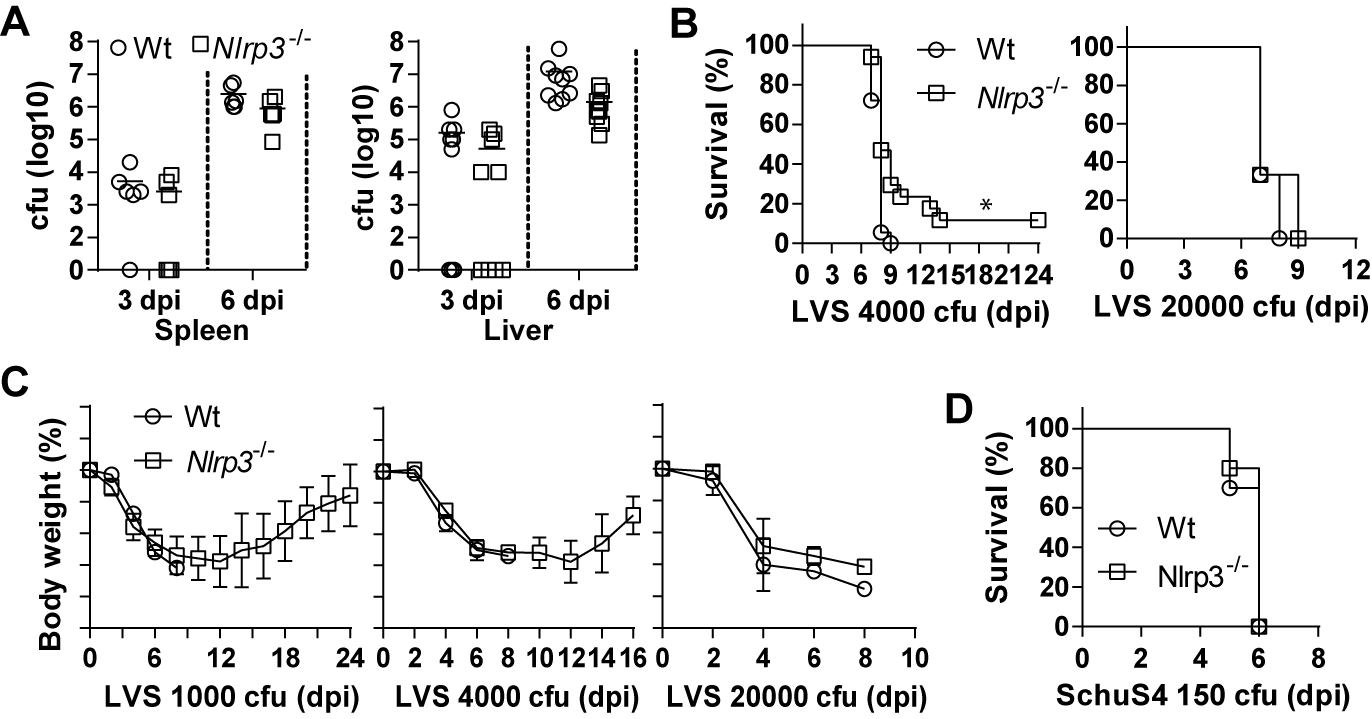

Supplement: S2 Fig — (A) Bacterial burden in spleen and liver following Ft LVS infection (mean ± SD of three independent experiments, n = 9, Student’s t-test). (B) Survival of mice following 4 LD100 and 20 LD100 Ft LVS (1000 cfu) infection (% survival of two independent experiments, n = 12, Log-rank (Mantel-Cox) test, *p<0.05) and. (C) Per cent body weight loss following Ft LVS infection (mean ± SD of three independent experiments). (D) Survival of mice following Ft SchuS4 (150 cfu) infection (% survival of two independent experiments, Log-rank test). (TIF) [file ppat.1006059.s002.tif]

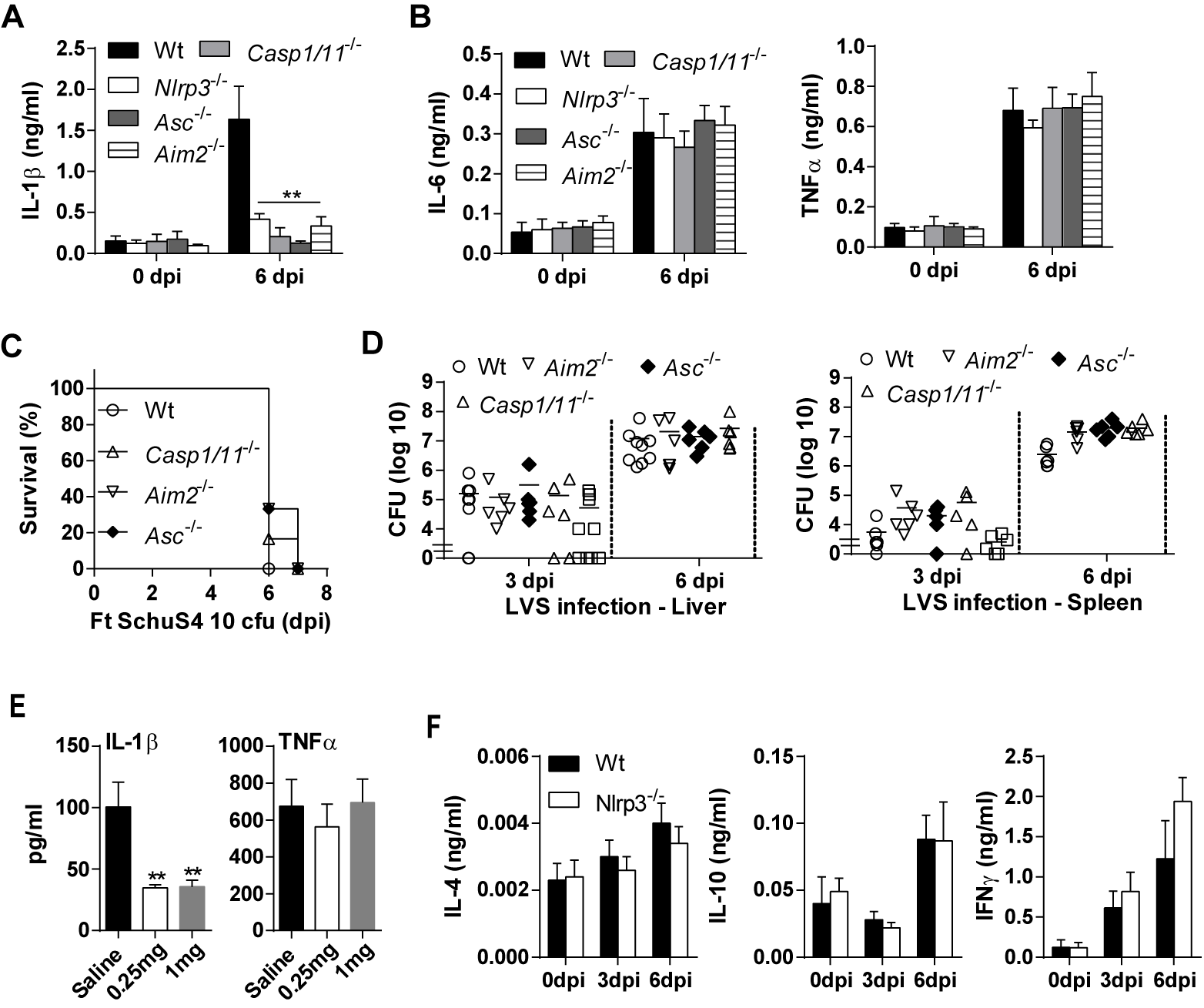

Supplement: S3 Fig — (A) Levels of IL-1β measured in lung homogenates at indicated days post-Ft SchuS4 infection (mean ± SD of three mice, Student’s t-test, **p<0.01 indicates difference from wildtype mice). (B) Levels of IL-6 and TNF measured in lung homogenates at indicated days post-Ft SchuS4 infection (mean ± SD of three mice, n = 6, Student’s t-test) (C) Survival of Ft SchuS4 (10 cfu) infected mice (% survival of two independent experiments, n = 10, Log-rank test). (D) Bacterial burden in spleen and liver following Ft LVS infection (mean ± SD of two independent experiments, n = 6, Student’s t-test). (E) Serum cytokine levels in LPS-injected (10 mg/kg bwt) mice treated with MCC950 (1 mg/mouse (50mg/kg bwt) or 0.25mg/mouse (12.5mg/kg bwt) daily at 2–7 dpi) (mean ± SD of three mice, Student’s t-test, **p<0.01). (F) Levels of IL-4, IL-10, and IFNɣ in lung homogenates at indicated days post Ft LVS infection (mean ± SD of 6 mice). (TIF) [file ppat.1006059.s003.tif]

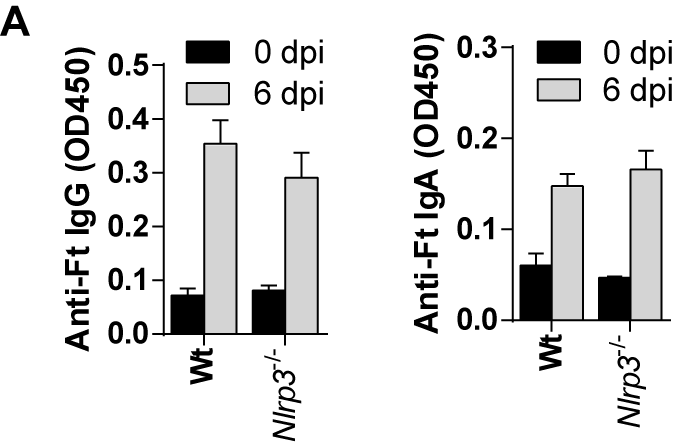

Supplement: S4 Fig — (A) Levels of anti-Ft IgG and IgA antibodies measured in BAL fluid of wildtype mice infected with Ft LVS (mean ± SD of OD450 from two independent experiments, n = 6, Student’s t-test). (TIF) [file ppat.1006059.s004.tif]

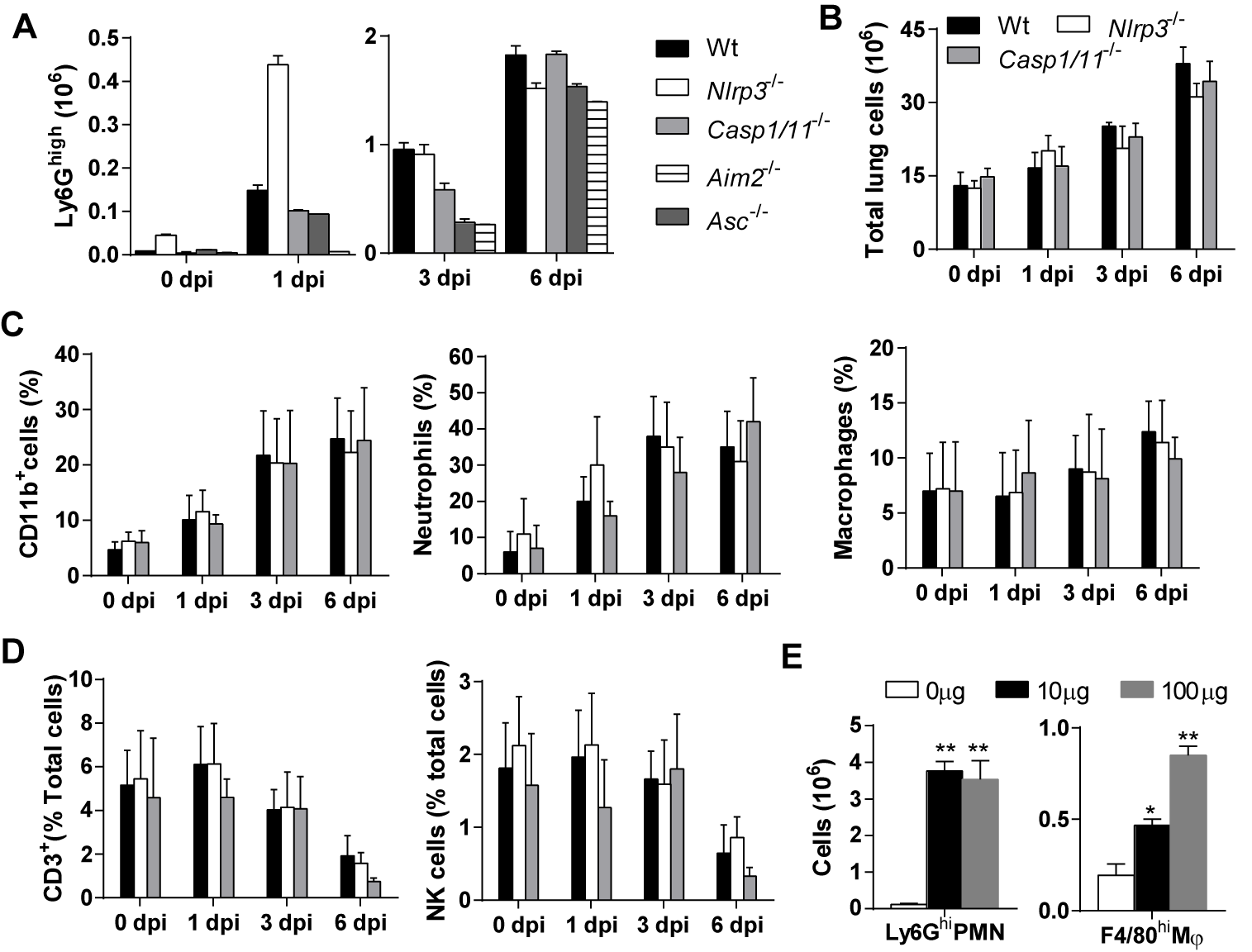

Supplement: S5 Fig — (A) Total numbers of Ly6G+ neutrophils in LVS-infected lungs (mean ± SD of two independent experiments). (B) Total numbers of cells recovered from LVS-infected lungs (mean ± SD of two independent experiments, n = 6, Student’s t-test). (C) Frequency of CD11b+ myeloid cells, Ly6G+ neutrophils, and F4/80+macrophages in LVS-infected lungs (mean ± SD of two independent experiments, n = 6, Student’s t-test. (D) Total numbers of CD3+ T cells and NK1.1+ cells in LVS-infected lungs (mean ± SD of two independent experiments, n = 6, Student’s t-test. (E) Total numbers of PMN and MØ in LPS-treated lungs after 48 hours without Ft LVS infection (mean ± SD of three mice, Student’s t-test, *p<0.05, **p<0.01). (TIF) [file ppat.1006059.s005.tif]

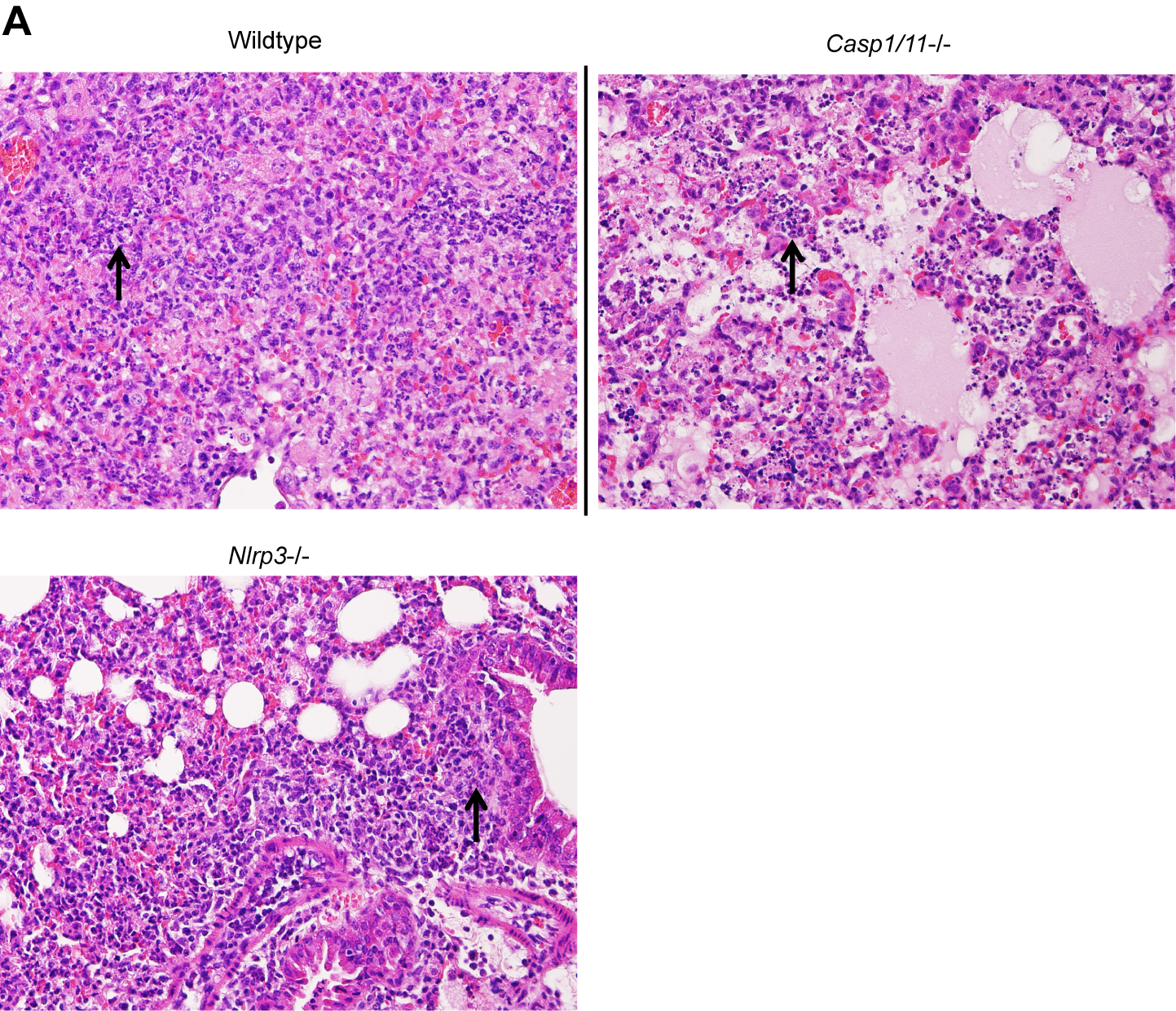

Supplement: S6 Fig — (A) Histological sections of lungs from LVS-infected mice at 6 dpi show inflammatory foci, massive necrosis and clumps of dead cells (arrow). Note abundant dead cells with degenerated nuclei as clumps (arrow) in lung sections from wildtype, Casp1/11-/- and Aim2-/- mice, while moderate numbers in Asc-/- mice and less number in Nlrp3-/- mice (HE, 400x). (TIF) [file ppat.1006059.s006.tif]

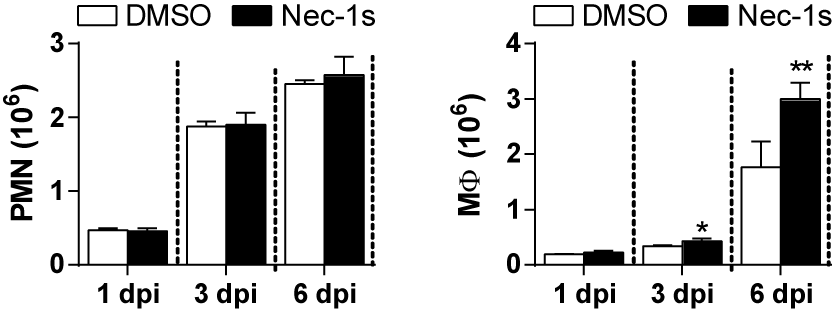

Supplement: S7 Fig — Total numbers of PMN and MØ in LVS-infected mice treated with DMSO or Nec-1s (mean ± SD of three mice, Student’s t-test, *p<0.05, **p<0.01). (TIF) [file ppat.1006059.s007.tif]
